# Phosphorylation of Extracellular Proteins in *Acinetobacter baumannii* in Sessile Mode of Growth

**DOI:** 10.3389/fmicb.2021.738780

**Published:** 2021-10-01

**Authors:** Sébastien Massier, Brandon Robin, Marianne Mégroz, Amy Wright, Marina Harper, Brooke Hayes, Pascal Cosette, Isabelle Broutin, John D. Boyce, Emmanuelle Dé, Julie Hardouin

**Affiliations:** ^1^Normandie Univ., UNIROUEN, INSA Rouen, CNRS, Polymers, Biopolymers, Surfaces Laboratory, Rouen, France; ^2^PISSARO Proteomic Facility, IRIB, Mont-Saint-Aignan, France; ^3^Infection and Immunity Program, Department of Microbiology, Monash Biomedicine Discovery Institute, Monash University, Melbourne, VIC, Australia; ^4^Université de Paris, CNRS, Laboratoire CiTCoM, Paris, France

**Keywords:** phosphorylation, *Acinetobacter baumannii*, biofilm, extracellular proteins, proteomics, Hcp, T6SS and pili

## Abstract

*Acinetobacter baumannii* is a problematic nosocomial pathogen owing to its increasing resistance to antibiotics and its great ability to survive in the hospital environment, which is linked to its capacity to form biofilms. Structural and functional investigations of post-translational modifications, such as phosphorylations, may lead to identification of candidates for therapeutic targets against this pathogen. Here, we present the first S/T/Y phosphosecretome of two *A. baumannii* strains, the reference strain ATCC 17978 and the virulent multi-drug resistant strain AB0057, cultured in two modes of growth (planktonic and biofilm) using TiO_2_ chromatography followed by high resolution mass spectrometry. In ATCC 17978, we detected a total of 137 (97 phosphoproteins) and 52 (33 phosphoproteins) phosphosites in biofilm and planktonic modes of growth, respectively. Similarly, in AB0057, 155 (119 phosphoproteins) and 102 (74 phosphoproteins) phosphosites in biofilm and planktonic modes of growth were identified, respectively. Both strains in the biofilm mode of growth showed a higher number of phosphosites and phosphoproteins compared to planktonic growth. Several phosphorylated sites are localized in key regions of proteins involved in either drug resistance (β-lactamases), adhesion to host tissues (pilins), or protein secretion (Hcp). Site-directed mutagenesis of the Hcp protein, essential for type VI secretion system-mediated interbacterial competition, showed that four of the modified residues are essential for type VI secretion system activity.

## Introduction

*Acinetobacter baumannii* is a Gram-negative nosocomial pathogen that mostly impacts patients in intensive care units and causes severe infections including pneumonia, bacteremia, endocarditis, skin and soft tissue infections, urinary tract infections, and meningitis (Bergogne-Bérézin and Towner, 1996; Dijkshoorn et al., 2007; Sengstock et al., 2010). This organism has been recently classified by the WHO as “Critical” (Priority 1, together with *P. aeruginosa* and *Enterobacteriaceae*) in the list of global priority antibiotic-resistant bacteria, for which the research and development of new and effective antibiotic treatments are urgently required. Several factors support this ranking (Shrivastava et al., 2018). This rapidly emerging pathogen is problematic worldwide owing to the dramatic increase of infections caused by multidrug-resistant (MDR), extensively drug resistant and even pan-drug resistant, isolates (Goic-Barisic et al., 2017; Lim et al., 2018). *A. baumannii* also has the capacity to survive for long periods of time in hospital settings, due to its considerable ability to form biofilms (Antunes et al., 2014), enabling it to survive desiccation (Gayoso et al., 2014), oxidative stress (Soares et al., 2010), or antiseptic treatments (Peleg et al., 2008). Thus, *A. baumannii* has an outstanding ability to adapt to detrimental environmental conditions.

Post-translational modifications (PTMs) are an important and effective strategy that bacteria use to adapt to environmental conditions. Phosphorylation of proteins is an important signaling event in prokaryotes (Mijakovic et al., 2016) and plays important roles in bacterial virulence, adaptation, and resistance (Stock et al., 2000; Dworkin, 2015; Standish et al., 2016; Tierney and Rather, 2019; Xie et al., 2019; Lipa and Janczarek, 2020). The reversible and dynamic PTMs include the addition or removal of phosphoryl groups by kinases and phosphatases, respectively, and can alter protein function by modulation of protein conformation, subcellular localization and protein-protein interactions (Watanabe and Osada, 2012).

Proteomics is a strategy that allows the global characterization of modified proteins in a complex sample and has been used to identify serine/threonine/tyrosine phosphoproteomes for many bacterial species (Yagüe et al., 2019). One study that analyzed the phosphoproteomes of two *A. baumannii* strains during growth in intracellular medium showed that there was twice as many phosphorylated residues in the highly invasive multidrug-resistant clinical isolate Abh12O-A2 compared to those in the reference strain ATCC 17978, suggesting that phosphorylation plays a key role in the regulation of drug resistance mechanisms (Soares et al., 2014).

Very few global proteomic studies have characterized PTMs of extracellular proteins (Ouidir et al., 2014a; Gaviard et al., 2019). However, this analysis is very important as virulence factors are often secreted and the presence of PTMs, like phosphorylation, can impact their function. Recently, analysis of extracellular proteins produced by *P. aeruginosa* identified multiple PTMs on a range of secreted virulence factors (Gaviard et al., 2019). Moreover, the PTM distribution on these virulence factors appeared to be different between the intra- and extracellular-sourced proteins. Therefore, it is important to characterize the PTMs of proteins produced by *A. baumannii* as these modifications are likely to contribute to the adaptability, virulence, and persistence of the organism.

Here, we have investigated, for the first time, the serine, threonine and tyrosine phosphoproteome in the extracellular medium sourced from *A. baumannii* in a biofilm mode of growth. Two strains were investigated: the reference strain ATCC 17978 and the virulent multi-drug resistant strain AB0057, isolated from a patient with an *A. baumannii* bloodstream infection. Overall, a total of 109 and 146 phosphorylated extracellular proteins were identified for strains ATCC 17978 and AB0057, respectively. We also showed that one specific phosphorylation site was essential for the activity of the *A. baumannii* type VI secretion system (T6SS). This study provides the first global phospho-secretome profiling of *A. baumannii* and represents a promising starting point for further investigations on the biological role of phosphorylation in *A. baumannii.*

## Materials and Methods

### Strains and Growth Conditions

For this study, two *A. baumannii* strains were used, ATCC 17978 (lacking the pAB3 plasmid, confirmed using PCR with previously described pAB3-specific primers) (Weber et al., 2015) and AB0057 (Hujer et al., 2006). Strains were grown overnight in Mueller Hinton Broth (MHB, Difco) at 37°C with shaking. Bacterial cultures were inoculated with approximately 1 × 10^7^ Colony Forming Units (CFU)/mL from overnight cultures. Biofilm cultures were grown in 500 mL of MHB containing 18.75 g of sterile glass wool (Merck) (Crouzet et al., 2014, 2017) and incubated at 37°C for 24 h with slow shaking (90 rpm). In parallel, planktonic cultures were grown in the same conditions (without glass wool) and incubated at 37°C for 24 h with vigorous shaking (140 rpm). Bacteria were harvested by centrifugation (8,000 × *g* for 15 min at 4°C), and supernatants containing extracellular proteins stored for analysis. For each condition, bacterial cultures were grown in triplicate.

### Secreted Protein Extraction

Each supernatant sample (500 mL) was filtered through a 0.22 μm membrane (GSWP 47 mm, Millipore) to remove residual bacteria. A protease/phosphatase inhibitor cocktail (50 μL, Halt^TM^ Protease and Phosphatase Inhibitor Single-Use Cocktail, EDTA-Free (100×), Thermo Scientific), and the histone deacetylase (HDAC) inhibitors nicotinamide (50 μL at 2 M, inhibitor of HDAC class III) and Trichostatin A (5 μL at 0.3 mM, inhibitor of HDAC classes I and II) (Sigma-Aldrich) were added to each sample. The secreted proteins were then concentrated to a volume of approximately 1 mL using Centricon plus-70 centrifugal filter units with a 10 kDa cutoff, according to the manufacturer’s protocol (Millipore). An ultracentrifugation step was applied to the concentrated samples (200,000 × *g*, 2 h, 4°C) to pellet outer membrane vesicles. The protein content of each supernatant representing extracellular proteins was then determined using the Bradford assay (Bio-Rad).

### Phosphopeptide Identification

Samples were prepared as described previously (Ouidir et al., 2014a, b). Briefly, extracellular protein samples (50 μg) were subjected to a short electrophoresis separation in an SDS-PAGE stacking gel (7%) then protein bands were excised, and the protein reduced with DTT, alkylated with iodoacetamide, before overnight digestion with trypsin (2 μg per band). Phosphopeptide enrichment was performed using metal oxide affinity chromatography (MOAC) with titanium dioxide beads (TiO_2_, Carlo Erba) as previously described (Ouidir et al., 2014a, b). The enriched phosphopeptides were then analyzed using a LTQ-Orbitrap Elite mass spectrometer coupled to an Easy nLC II system or using Qexactive Plus coupled to an Ultimate 3000 (all from Thermo Scientific). Raw data files were processed using Proteome Discoverer 1.4 software (Thermo Scientific). Peak lists were searched using the MASCOT search software (Matrix Science) against the *A. baumannii* ATCC 17978 and *A. baumannii* AB0057 databases, containing 4097 and 4118 protein sequences, respectively.^1^ The false discovery rate (FDR) threshold for identifications was set at 1% (for proteins and peptides). For each identification, a peptide ion score higher than 14 for phosphorylation; a peptide rank of 1, a *q*-value and an expectation value below 0.05 was considered significant. To avoid biased automatic annotation, all phosphopeptide spectra were manually inspected. For the localization of the phosphosites, the probability results obtained from MASCOT and phosphoRS (Thermo Scientific) were used. MS proteomics data have been deposited to the ProteomeXchange Consortium via the PRIDE partner repository with the data set identifier. Our project accession in PRIDE is PXD027208.

### Bioinformatic Analysis of Modified Proteins

Proteins were manually classified on the basis of predicted biological function using information gained from the annotated function, Uniprot^2^ and/or other available information (Resistome, VFDB experimentally demonstrated, Virulence Finder, *E. coli* main virulence genes, Integron and Macromolecular Systems) in the Genoscope databases (see text footnote 1; Vallenet et al., 2020). For hypothetical proteins, we used BLAST2Go 5.2.5 software, an automated functional annotation tool, for identification of (i) similar or potentially homologous sequences from NCBI database (NCBI-nr), (ii) protein domains and families from Interpro databases (EMBL-EBI), and (iii) Gene Ontology (biological process, molecular function). The Kyoto Encyclopedia of Genes and Genomes (KEGG) was used to map the pathways. STRING database (version 11.0) was used to investigate protein-protein network interactions with a high confidence score (0.7). WebLogo 2.8.2^3^ and Icelogo^4^ were used with default parameters to visualize conserved patterns in sequence motifs (15 amino acids upstream and downstream of the phosphorylation site). Subcellular localization of the phosphoproteins, corresponding to the phosphopeptides identified, was predicted by PSORTb version 3.0.2 (Yu et al., 2010). To further characterize proteins, multiple bioinformatics tools were employed including SignalP 5.0^5^ and TatP 1.0,^6^ to determine signal peptides of proteins predicted to be secreted by Sec and Tat translocons (Bendtsen et al., 2004, 2005b), LipoP^7^ to predict putative lipoproteins in the outer membrane by searching for lipoboxes (Juncker et al., 2003), and Secretome 2.0^8^ and MatureP,^9^ to predict secreted proteins (Bendtsen et al., 2005a; Orfanoudaki et al., 2017). MoonProt^10^ was used to find putative moonlighting proteins, defined as secreted proteins that have other known functions (Chen et al., 2018). BlastP^11^ was used to interrogate the Protein Data Bank (PDB) and structures to identify those with high sequence identity to the identified proteins. All figures representing three-dimensional structures were drawn with Pymol (Delano, 2002).

### Generation of an AB307-0294 *hcp* Mutant and Complementation Strain

The AB307-0294 *hcp* mutant was generated using splice overlap extension (SOE) PCR and allelic exchange (Adams et al., 2008; Aranda et al., 2010; Tucker et al., 2014; see “Supplementary Materials and Methods” for full details). Briefly, a SOE PCR product consisting of a kanamycin cassette (containing *neo*), flanked by Flp recombinase Recognition Target (FRT) sites and regions representing upstream and downstream of *hcp*, was introduced into *A. baumannii* AB307-0294 via electroporation (Choi et al., 2006). Selected kanamycin resistant *hcp* mutants were provided with the plasmid pAT03 and grown in the presence of isopropyl-β-d-1-thiogalactopyranoside (IPTG) to induce Flp recombinase expression and excise the kanamycin cassette from the genome. The integrity of the markerless Δ*hcp* mutant was confirmed by PCR and DNA sequencing. For complementation, an intact copy of *hcp*, including the native ribosome binding site, was PCR-amplified and cloned into the pBASE expression plasmid (Supplementary Table 2). The *hcp* complementation plasmid and empty pBASE were then used to separately transform the AB307-0294 *hcp* mutant, generating strains AL3844 (Δ*hcp*[pBASE]) and AL3942 (Δhcp[*hcp*]).

### Mutation of Individual Hcp Phosphosites in *A. baumannii* Strain AB307-0294

To assess the role of specific amino acids in Hcp function, the Δ*hcp* mutant was separately provided with a range of plasmids, each expressing *hcp* with specific mutations to produce a single amino change in the protein (S18A, S18D, S31A, S41A, T43A, or S44A changes) compared to the wild-type Hcp. Each change to *hcp* was generated using PCR or SOE-PCR with the appropriately designed primers. PCR fragments were then cloned into pBASE (see “Supplementary Materials and Methods” for full details).

### Detection of the T6SS Protein Hcp Using Western Immunoblotting

Production and secretion of the Hcp protein in *A. baumannii* was assessed by subjecting whole-cell lysates (WCL) and culture supernatants to western immunoblotting using anti-Hcp polyclonal sera, as described previously (Fitzsimons et al., 2018) but with minor modifications (see “Supplementary Materials and Methods” for full details). The intensity of each immunoreactive band was quantified from the peak area of histograms generated using ImageJ software (Schneider et al., 2012). Significance of differences in Hcp production were assessed using one-way analysis of variance (ANOVA; GraphPad Prism, version 9) with Tukey’s multiple-comparison post-test; a *P* value of <0.05 was accepted as statistically significant.

## Results and Discussion

The ability of *A. baumannii* to form biofilms significantly threatens human health as it enhances the ability of this pathogen to persist and survive in response to desiccation and various stresses and for prolonged periods in hospital environments. In order to identify phosphorylation events that occur during biofilm growth and determine those which may play important roles in bacterial virulence or resistance, we investigated the phospho-secretome of AB0057 in planktonic and biofilm modes of growth. We also assessed the @ @ phospho-secretome of ATCC 17978 pAB3^–^ strain, known to have an active T6SS and to produce biofilms (Weber et al., 2015; Moon et al., 2017).

### Phospho-Secretome Analysis of *A. baumannii* Strains ATCC 17978 and AB0057

Using a large-scale proteomic approach, we first assessed the phosphorylation of serine, threonine and tyrosine residues on extracellular proteins (S/T/Y phosphoproteomes) derived from biofilm and planktonic growth of *A. baumannii* reference strain ATCC 17978 and the virulent isolate AB0057. After bacterial culture, extracellular proteins were digested and phosphorylated peptides were enriched using titanium dioxide (TiO_2_), as described previously (Ouidir et al., 2014a). Phosphopeptides were then analyzed by nanoLC-MS/MS. Following peptide identification, all data were manually checked to confirm the best mass spectrum interpretation and peptide sequence and localization of the PTM. If localization of the phosphorylation site was not possible then the putative modified residues were annotated in brackets.

**FIGURE 1:**
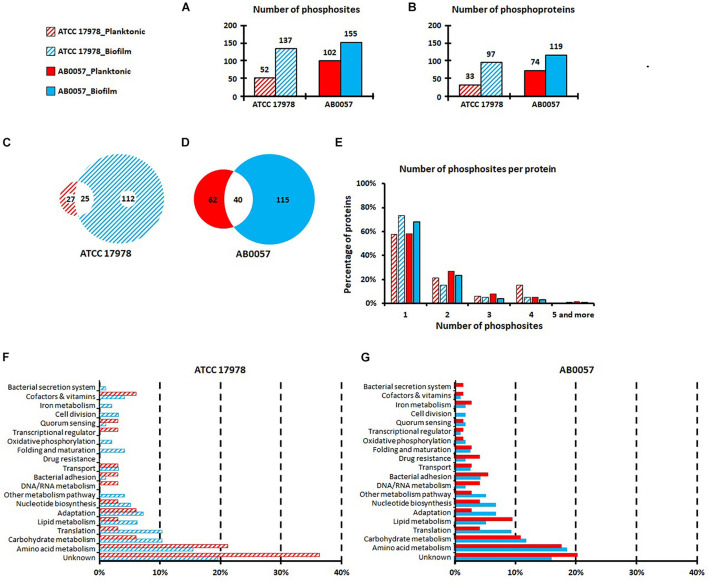
Numbers of phosphosites **(A)** and phosphoproteins **(B)** identified in *A. baumannii* ATCC 17978 and AB0057 strains in planktonic and biofilm modes of growth. **(C,D)** Phosphosite distribution between planktonic and biofilm modes of growth for ATCC 17978 and AB0057, respectively. **(E)** Number of phosphosites per protein in planktonic and biofilm modes of growth for ATCC 17978 and AB0057. **(F,G)** Functional categories of the phosphorylated proteins in planktonic and biofilm modes of growth for ATCC 17978 and AB0057, respectively. Hatched red: ATCC 17978 in planktonic. Hatched blue: ATCC 17978 in biofilm. Red: AB0057 in planktonic. Blue: AB0057 in biofilm.

For both strains, the overall number of phosphorylated sites was higher in peptides derived from the biofilm mode of growth than from the planktonic mode of growth. Indeed, 137 modified sites (corresponding to 124 unique peptides and 97 unique proteins) in ATCC 17978 and 155 modified sites (corresponding to 144 unique peptides and 119 unique proteins) in AB0057 were identified in biofilm, while 52 phosphosites (44 unique peptides and 33 unique proteins) in ATCC 17978 and 102 phosphosites (92 unique peptides and 74 unique proteins) in AB0057 were identified in planktonic mode (Figures 1A,B and Supplementary Tables 3–10). More phosphorylated sites were identified in the peptides derived from the virulent strain AB0057 than in peptides from the reference strain ATCC 17978, especially in the planktonic growth samples. Interestingly, less than half of the phosphosites identified in the peptides derived from planktonic grown cells were also present in peptides derived from biofilm grown cells (Figures 1C,D).

No significant difference was observed in the phosphorylation distribution onto S/T/Y (Supplementary Table 9). To identify conserved motifs, sequences within 15 amino acids upstream and downstream of the phosphorylated sites were examined for similarity. However, no consensus motif surrounding each of the phosphorylated sites was found.

The majority of proteins were phosphorylated at only one site (Figure 1E). In strain AB0057, elongation factor Tu (AB57_0362) had the highest number of phosphorylations [six phosphorylations on T17, (T26/T27), Y77, Y88, T94, and T109]. In strain ATCC 17978, five phosphorylated sites were identified in the T6SS tube protein Hcp (S18, S31, S41, T43, and S44) but these additions were only present on extracellular Hcp isolated from biofilm cultures (Supplementary Table 8).

Initial bioinformatic analyses indicated that the majority of the modified proteins were predicted to be cytoplasmic, with only 3% predicted to be secreted (Supplementary Tables 7, 8). However, it is well known that many predicted cytoplasmic proteins can also be secreted, e.g., DnaK (Karched et al., 2019), EF-Tu (Chiu et al., 2017), and GroEL (Jeffery C.J., 2018). Such multifunctional proteins are also known as moonlighting proteins and may be involved in adhesion to the host or in microbial pathogenesis (Hagemann et al., 2017; Harvey et al., 2019; Jeffery C.J., 2019; N’Diaye et al., 2019). Nine phosphoproteins in ATCC 17978 and 10 phosphoproteins in AB0057 were identified as putative moonlighting proteins with adhesion or binding activities (Supplementary Tables 7, 8). The sequences of all phosphorylated proteins were analyzed using a number of bioinformatic tools. In total, 23 and 29 proteins with signal peptides were identified in ATCC 17978 and AB0057, respectively, and are therefore predicted to be secreted by the Sec pathway. Using SecretomeP, 36 proteins in ATCC 17978 and 35 proteins in AB0057 were predicted to be secreted *via* a non-classical secretion pathway (Kang and Zhang, 2020). MatureP identified 29 proteins in ATCC 17978 and 46 proteins in AB0057 secreted by the Sec secretory system. Furthermore, 12 lipoproteins were identified (five in ATCC 17978 and seven in AB0057). Taking into consideration the data from all of these analyses, the final number of predicted secreted proteins (estimated to be 3% using initial bioinformatic analysis) was determined to be 38%.

The phosphorylated proteins identified were classified according to the classical functional categories (Figures 1F,G). The majority of planktonic and biofilm derived extracellular proteins were classified into the amino acid metabolism group and the unknown function group, some of which in the latter group may potentially contribute to virulence by currently unidentified mechanisms. Interestingly, compared to ATCC 17978 planktonic growth, there were more ATCC 17978 biofilm-derived extracellular proteins classified into bacterial secretion system, iron metabolism, oxidative phosphorylation and folding and maturation functional groups (Figure 1F). For the clinical isolate AB0057, compared to planktonic growth, there were more biofilm-derived extracellular proteins classified into the adaptation protein functional group (Figure 1G). Furthermore, differences in the distribution of the identified proteins into functional groups were clearly noted between the two strains for the secreted proteins in the following categories: DNA/RNA metabolism, transcriptional regulation, and drug resistance.

### Functions of Selected Phosphorylated Proteins

#### Type VI Secretion System: Phosphorylation of Hcp

**FIGURE 2:**
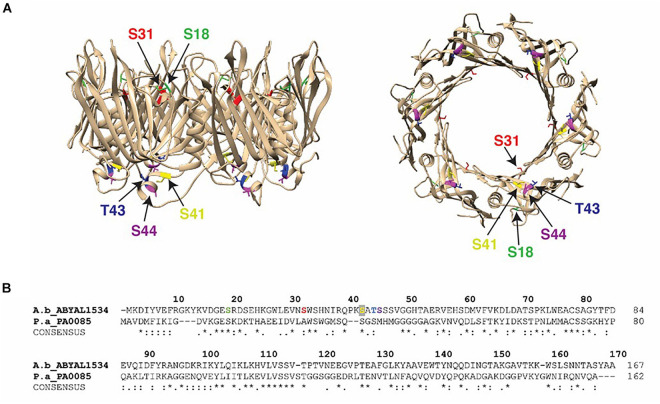
Hcp phosphorylation sites: **(A)** Crystal structure of the *A. baumannii* Hcp (PDB 4W64) with specific phosphosites colored (S18, light green; S31, red; S41, yellow; T43, blue; S44, purple) and the Hcp hexameric donut structure viewed from the side (left) and bottom (right). **(B)** Alignment of Hcp protein from *A. baumannii* ATCC 17978 (A.b_ABYAL1534) and *P. aeruginosa* (P.a_PA0085). Completely invariant residues are indicated with an asterisk (*), highly conserved regions are marked with a colon (:) and semi-conserved residues are dotted (.). Identified peptides and phosphosites in our study are colored and designated by arrows.

The T6SS is a multiprotein apparatus that delivers protein effectors into both prokaryotic and eukaryotic cells (Pukatzki et al., 2009; Ho et al., 2017). In various Gram-negative species it plays a critical role in biofilm formation (Aschtgen et al., 2008) and as a defensive weapon in inter-bacterial competition and/or attack of host cells (Schwarz et al., 2010; Russell et al., 2011; Basler et al., 2013; Carruthers et al., 2013). In the *A. baumannii* strains AB0057 and ATCC 17978, the genes encoding the main structural components of the T6SS apparatus are encoded within one, well conserved, locus composed of 18 and 22 genes, respectively. Furthermore, three distinct VgrG loci are located elsewhere on the genome. Each VgrG locus encodes the VgrG tip protein, and a cognate effector protein and a cognate immunity protein. Hcp proteins form the needle of the T6SS via the stacked formation of multiple donut-shaped Hcp hexamers. In some bacterial species Hcp can act as a specialized effector or as a transporter and/or chaperone of T6SS cargo effectors (Ruiz et al., 2015). The presence of Hcp in the cell culture supernatant is a molecular marker of a functional T6SS (Mougous et al., 2006; Pukatzki et al., 2009; Weber et al., 2013; Corcionivoschi et al., 2015). Hcp proteins (AB0057_1481 and ABYAL1534) were detected in both planktonic and biofilm modes of growth, suggesting a functional T6SS is produced by both strains under both growth conditions (Supplementary Table 10). In agreement with the literature (Weber et al., 2015), Hcp is secreted by ATCC 17978 lacking the plasmid pAB3 (encoding a TetR repressor) at greater amounts when compared to secretion by strain AB0057. Interestingly, the five phosphosites (S18, S31, S41, T43, and S44) on Hcp were only detected in biofilm mode of growth for ATCC 17978 (Supplementary Tables 6, 8). Examination of the predicted 3D model of the *A. baumannii* Hcp hexamer indicates that these phosphosites are positioned at the top and the bottom of the donut-shaped structure (Figure 2).

**FIGURE 3:**
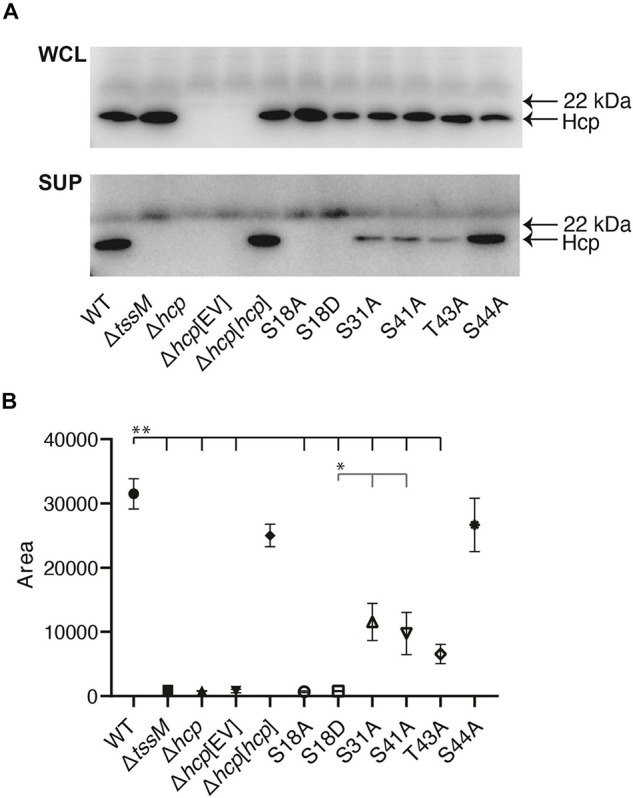
Role of different phosphosites in Hcp secretion. Analysis of Hcp secretion by various *A. baumannii* AB307-0294 strains using western immunoblotting with Hcp-specific antiserum. **(A)** Whole-cell lysates (WCL) and secreted samples (SUP) from the wild-type AB307-0294 (WT), the Δ*tssm* mutant, Δ*hcp* mutant, Δ*hcp* mutant transformed with empty vector Δ*hcp*[EV], Δ*hcp* mutant complemented with an intact copy of *hcp* (Δ*hcp*[*hcp*]) and the Δ*hcp* mutant transformed with the expression constructs S18A, S18D, S31A, S41A, T43A, and S44A. The arrows at the right indicate the position of the 22 kDa MW Myoglobin Red protein (SeeBlue^TM^ Plus2 pre-stained protein standard) and the predicted position of the Hcp protein. Images are representative of three biological replicates. Quantitative analysis of the amount of secreted Hcp protein present for each strain was determined by densitometry of triplicate biological replicate western blots **(B)**. Symbols represent the mean of the quantified peak area of obtained band intensity histograms for each strain ± standard deviation of the means. **, *P* < 0.01, *, *P* < 0.05.

To test the importance of the Hcp phosphosites for T6SS function, we mutated selected sites in a recombinant version of Hcp derived from *A. baumannii* strain AB307-0294 (l00% amino acid identity with ATCC 17978 and AB0057 Hcp). Individual amino acid substitutions of the identified Hcp phosphosites were performed with alanine, a non-phosphorylable amino acid. Each modified *hcp* gene was cloned into the expression plasmid pBASE and introduced into an *A. baumannii* AB307-0294 *hcp* mutant, and T6SS function in each strain assessed *via* the immunodetection of Hcp secretion. An S18A amino acid substitution in Hcp abolished Hcp secretion via T6SS activity as no Hcp was detected in the supernatant (Figure 3). Similarly, an S18D phosphomimetic substitution in Hcp also resulted in no Hcp secretion. In contrast, Hcp proteins with S31A, S41A, T43A, or a S44A substitution were functional as T6SS-secreted Hcp was detected in the supernatant, though levels of secreted Hcp were significantly reduced for Hcp proteins with S31A, S41A, and T43A substitutions, compared to mutant provided with wild-type Hcp or Hcp S44A. In all strains provided functional copy of wild-type or mutated *hcp*, Hcp protein was detected in the whole cell lysate, indicating that each substitution had no effect on the production of Hcp (Figure 3). The TssM protein is an essential component of the T6SS membrane-stabilizing structure. The Δ*tssM* strain was used as an additional negative control in the immunoblot as this mutant cannot secrete Hcp due to lack of a functional T6SS (Fitzsimons et al., 2018).

In *P. aeruginosa* PAO1, Hcp protein not only forms the T6SS tube but also associates with effectors, protects them from proteolysis, and likely traffics with effectors during transport (Silverman et al., 2013). Specific amino acids in Hcp, such as S31 or L28, can inhibit or affect the ability of the protein to secret the associated effector. Thus, T6SS effectors form a unique network of interactions with Hcp. In *Francisella tularensis*, the importance of the phosphorylation of the T6SS sheath protein IglB was recently highlighted (Ziveri et al., 2019). A Y139 mutation in IglB was able to abrogate the T6SS assembly regardless of the substituted residue (Y139A, Y139D, or Y139E). The phosphorylation status of Y139 therefore modulates the dynamics of T6SS assembly. Our results indicate that the phosphorylation of Hcp amino acid S18 seems to play a pivotal role in *A. baumannii* Hcp secretion via an active T6SS. These results highlight the importance of the characterized phosphorylated residues, especially S18, on T6SS activity in *A. baumannii.*

#### Adhesion Process: Phosphorylation of Fimbrial Subunits

In *A. baumannii*, type I pili represent a major class of surface appendage mediating bacterial attachment to host cells or abiotic surfaces and are required for biofilm formation (Tomaras et al., 2003; Álvarez-Fraga et al., 2016; Wood et al., 2018; Di Venanzio et al., 2019). Each pilus consists of self-polymerizing pilin subunits and in some instances a distal specialized subunit called the tip pilin. Pilin is also the main protein recovered from the *A. baumannii* biofilm matrix (Nait Chabane et al., 2014). Pili are assembled *via* a chaperone-usher pathway (CUP) that involves a periplasmic chaperone and an outer membrane usher for secretion to the cell surface (Tomaras et al., 2003; Zavyalov et al., 2010; Busch and Waksman, 2012; Garnett et al., 2012; Bao et al., 2013; Berry et al., 2014; Werneburg and Thanassi, 2018). One of the most well-studied type 1 pili is the Csu system (Tomaras et al., 2003; Pakharukova et al., 2018). The Csu pilus is made of four subunits, CsuA/B (the major pilin subunit), CsuA, CsuB, and the tip subunit CsuE, which uses three distal hydrophobic loops to interact with hydrophobic substrates (Pakharukova et al., 2018). The CsuC and CsuD proteins are the chaperone and the usher components of this system, respectively (Pakharukova et al., 2018). Analysis of *A. baumannii* strains ATCC 17978 and AB0057 detected phosphorylations on the major subunit CsuA/B (Supplementary Tables 7, 8 and Supplementary Figure 1) within the N-terminus region known to be involved in the interaction with the chaperone and in subunit polymerization. Indeed, assembly of each pilus starts in the periplasm with stabilization of a subunit by the chaperone. This occurs *via* a donor strand complementation process, where the β-strand G1 of the chaperone induces an immunoglobulin-like fold of the subunit (Choudhury et al., 1999; Sauer et al., 1999). Following folding, a donor β-strand exchange mechanism occurs where the G1 β-strand is displaced by the N-terminal extension (donor strand Gd) of an incoming subunit (Remaut et al., 2006; Allen et al., 2012). The phosphorylation of S36 (in AB0057) and S38 (in ATCC 17978) was detected in the CsuA/B subunit proteins produced by both strains. These residues are located in the region immediately following the end of the Gd β-strand (Supplementary Figure 1). It was previously shown that mutation of the P5 residue (I37A mutant) leads to a drastic decrease of CsuA/B polymerization (Remaut et al., 2006; Pakharukova et al., 2015). Our finding in *A. baumannii* suggests phosphorylations could decrease the accommodation of the Gd β-strand into the groove of a neighboring subunit and thus impact polymerization. Other phosphorylations were identified in the subunit A strand (T39, T42, and S46), that can be hydrogen bonded to the A1 β-strand of the CsuC chaperone during the formation of a high-energy folding intermediate and before pilus polymerization and translocation (Pakharukova et al., 2015). Overall, these modifications could regulate CsuA/B interactions with the CsuC chaperone and contribute to the stabilization of its soluble form.

In addition, phosphorylations were also detected on the main subunits (FimA-like) of two other CUP pili in strain AB0057. The genes for these CU systems are located in two loci, each encoding four proteins (Supplementary Table 7) as follows: the FimA-like main pilin (AB57_1747 and AB57_2007 have 30 and 28% identity, respectively, with the *E. coli* FimA), the PapD chaperone (AB57_1746 and AB57_2006), the PapC outer membrane usher (AB57_1745 and AB57_2005) and the tip adhesion pilin (AB57_1744 and AB57_2004). These CUP pili were shown to be involved in biofilm formation on solid supports and in bacterial attachment to A549 epithelial cells (Rumbo-Feal et al., 2013; Álvarez-Fraga et al., 2016). As shown by the sequence alignment of these subunits with FimA proteins from different species (Supplementary Figure 2), one phosphorylation is commonly localized on the B” β-strand of both subunits, before the second cysteine necessary for the formation of a disulfide bond, and this residue is conserved in all the aligned pili subunits (Puorger et al., 2011; Zyła et al., 2019). Interestingly, the B” β-strand (T45-C58) corresponds to a sequence required for targeting FimA monomers to mitochondria (Sukumaran et al., 2010; Zyła et al., 2019). Indeed, previous studies demonstrated that soluble FimA monomers from invasive bacterial pathogens (*E. coli* K1, *Salmonella* or *Shigella*) may target the VDAC-hexokinase complex at mitochondria to delay host cell apoptosis during the early phase of infection (Sukumaran et al., 2010). As *A. baumannii* is able to invade epithelial cells via a zipper-like mechanism and persist in these cells (Choi et al., 2005, 2008; Parra-Millán et al., 2018), FimA-like subunits may possess a similar function and phosphorylation could be an important event to modulate pili subunit-mitochondria interactions.

The tip pilins of both CUP systems, AB57_2004 and AB57_1744, shared 36 and 35% identity, respectively, with the adhesin MrkD_1P_ of *Klebsiella pneumoniae* (PDB code 3U4KA). MrkD_1P_ mediates adherence to type V collagen through different sites identified by site-directed mutational analysis (Schurtz Sebghati et al., 1998; Rêgo et al., 2012). Interestingly, the phosphorylated residues T59, T61 and T176 are well conserved and located close to sites directly involved in type V collagen binding (Supplementary Figure 3). These data suggest that phosphorylation of these residues may play a role in interactions with host tissues. Taken together, these identified phosphorylations may play important roles in CU pili biogenesis or directly in link with eukaryotic cells interactions.

### Drug Resistance: Phosphorylation of β-Lactamases

**FIGURE 4:**
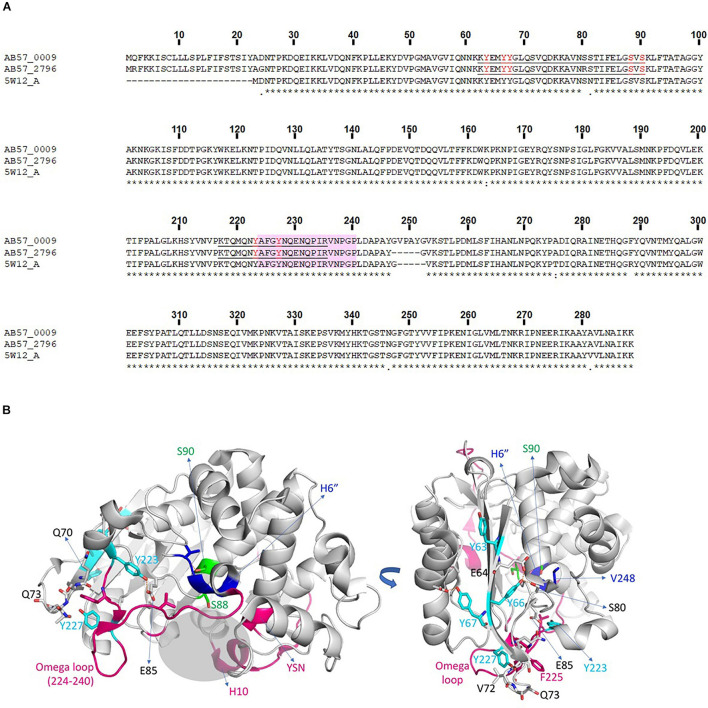
β-lactamase phosphorylations. **(A)** Alignment of ADCs from *A. baumannii* AB0057 (AB57_0009 and AB57_2796) with β -lactamase 5W12-A in the PDB. Completely invariant residues are indicated with an asterisk (*), highly conserved regions are indicated with a colon (:) and semi-conserved residues are indicated with a dot (.). Identified peptides and phospho-sites in our study are underlined and in red. **(B)** Structural representation of a β-lactamase from *A. baumannii* solved in complex with an inhibitor (PDB code 5W12). The phosphoY residues are colored in cyan and the phosphoS residues in green. The residues interacting with the modified Y or S are represented in gray sticks, and the interaction interface is indicated by brown dotted lines. The additional five amino acids present in the AB57_0009 protein are located in the blue zone in the H6” helix. Structural regions flanking the binding site, carrying mutations involved in increased resistance, are colored in magenta. The binding site is shown with a gray shaded area. The structural regions lining the binding site, bearing the mutations implicated in an increase of resistance, are colored in magenta. The binding site is materialized as a gray shaded area.

The AB0057 *A. baumannii* strain is a multidrug resistant clinical isolate; it is particularly resistant to β-lactams including carbapenems due to the presence of several β-lactamases (Hujer et al., 2006). We detected several phosphorylations on different β-lactamases produced by AB0057 (Supplementary Table 7). We observed four phosphosites [(Y63/Y66/Y67), S88, S90 and (Y223/Y227)] on ADC (*Acinetobacter*-derived cephalosporinase) proteins, AB57_0009 and AB57_2796. As these two proteins share 97.68% identity, it is not possible to precisely assign all of the phosphorylation sites to one or the other protein. However, phosphosites S88 and S90 were unambiguously assigned to the AB57_0009 protein. Residue S88 corresponds to the catalytic serine of the enzyme, making a covalent bond with the antibiotic before hydrolysis of the β-lactam, and S90 is oriented toward the small helix H6” that follows the Ω loop, shown to be extended of five amino acids in the AB57_0009 protein compared to other members of the class C β-lactamases. The phosphoY sites were located in the Ω loop [(Y223/Y227)] and the β-strand [(Y63/Y66/Y67)] (Figure 4), those in the latter being involved in the structural stability (Caselli et al., 2018). The Ω loop, localized at the entrance of the enzyme active site, is involved in the catalytic cycle of the β-lactam hydrolysis and most of identified mutations in this loop alter the susceptibility of the bacterium to β-lactam antibiotics.

For the β-lactamase AB57_0551, we identified one phosphosite [S79/T80] corresponding to the catalytic serine residue S79. This protein shares 99% amino acid identity with the class-D β-lactamase OXA-23 whose structure has been solved in complex with a molecule of meropenem linked covalently to the catalytic serine (PDB code 4JF4) (Harper et al., 2018).

In agreement with these results, a link between phosphorylation and the modulation of β-lactamase activity was recently reported. Lai et al. (2016) showed the importance of the S90 phosphosite in the catalytic motif S^88^VS^90^K of AmpC (ADC) from *A. baumannii* SK17, corresponding to the phosphoS90 found in AB57_0009. The phosphoablative S90A mutant had higher β-lactamase activity and increased resistance to imipenem, in contrast to the phosphomimetic S90D and S88D mutants which were more sensitive to imipenem. Thus, phosphorylation in the catalytic site region could repress enzyme activity. It should be also noted that phosphorylation of S90 (AB57_0009) and [S79/T80] (AB57_0551) were exclusively detected in extracellular proteins derived from planktonic growth. Taken together, these data suggest that during planktonic lifestyle, *A. baumannii* AB0057 would be more sensitive to antibiotics such as carbapenems.

### Secondary Functions: Phosphorylation of Moonlighting Proteins

Moonlighting proteins are proteins that can have two or more distinct biochemical functions (Jeffery C., 2019). They increase the complexity of bacterial regulation and the specific function of each depends on cellular localization, cellular ligands, and/or substrates. In this current study, 39 moonlighting phosphoproteins were identified, including DnaK, EF-Tu, GroEL, and peroxiredoxin (Supplementary Tables 7, 8). Thirteen of these proteins had been assigned a second role in adhesion (to plasminogen, eukaryotic cells, fibronectin), virulence or modulation of host immune response (Supplementary Table 11; Das et al., 2010; Henderson and Martin, 2013; Jeffery C., 2018). EF-Tu functions at the ribosomal level, but in *P. aeruginosa* it is also exported and binds to plasminogen (Kunert et al., 2007) and in *M. pneumonia* it has been shown to bind to fibronectin (Dallo et al., 2002). In *Bacillus cereus*, EF-Tu can be recruited onto the bacterial surface and act as an environmental sensor to detect the principal skin neuropeptide, substance P (N’Diaye et al., 2019). *A. baumannii* EF-Tu from both ATCC 17978 and AB0057 contained many phosphorylated sites. As all of the moonlighting proteins were phosphorylated on serine, threonine or tyrosine, it can be envisaged that phosphorylation can act as a switch to modulate protein function or protein secretion by either the addition of negative charge or via steric hindrance.

## Conclusion

Due to the crucial role of phosphorylation in bacterial virulence, resistance or adaptation, phosphorylation mapping is an important prerequisite for the discovery of novel regulatory mechanisms. Investigation of protein phosphorylation is essential to understand its role in bacterial physiology and to identify new control strategies for combatting the spread of MDR bacteria. In this study, we used the reference strain ATCC 17978 and the clinical isolate AB0057 to investigate for the first time the phospho-secretome of *A. baumannii* when in the biofilm mode of growth, which promotes bacterial environmental persistence and antibiotic tolerance. The comparative study of planktonic with biofilm modes of growth showed an enhanced number of phosphorylated proteins in cells grown within a biofilm. In addition, a higher number of phosphorylation events was observed in proteins derived from the virulent and antibiotic resistant strain AB0057, compared to those identified in the reference strain ATCC 17978.

Our data show a pivotal role of phosphorylation in many biological processes, including antibiotic resistance, biofilm formation and virulence. Indeed, phosphorylation of residues located within the catalytic site of several β-lactamases indicates this activity modulates their enzymatic activity. Phosphorylation of pili proteins could modulate polymerization, contribute to the stabilization of their soluble form, and could play a role in pili-facilitated adhesion to eukaryotic cells. Many phosphosites were detected on the T6SS tube protein Hcp and we showed that phosphorylation at amino acid S18 is required for Hcp secretion and therefore T6SS activity. Phosphorylation at three other sites on Hcp also contributes significantly to T6SS activity. This work is a promising starting point for further investigations into the biological role of phosphorylation on secreted proteins during growth in biofilms.

## Data Availability Statement

The datasets presented in this study can be found in online repositories. The names of the repository/repositories and accession number(s) can be found in the article/Supplementary Material.

## Author Contributions

ED, JB, and JH contributed to conception and design of the study. SM, BR, MM, AW, MH, BH, and IB performed the experiments. SM, MM, MH, IB, JB, ED, and JH wrote the manuscript. All authors contributed to manuscript revision, read, and approved the submitted version.

## Conflict of Interest

The authors declare that the research was conducted in the absence of any commercial or financial relationships that could be construed as a potential conflict of interest.

## Publisher’s Note

All claims expressed in this article are solely those of the authors and do not necessarily represent those of their affiliated organizations, or those of the publisher, the editors and the reviewers. Any product that may be evaluated in this article, or claim that may be made by its manufacturer, is not guaranteed or endorsed by the publisher.

## Footnotes

1
http://www.genoscope.cns.fr

2
http://www.uniprot.org/

3
https://weblogo.berkeley.edu/

4
https://iomics.ugent.be/icelogoserver/

5
http://www.cbs.dtu.dk/services/SignalP/

6
http://www.cbs.dtu.dk/services/TatP/

7
http://www.cbs.dtu.dk/services/LipoP/

8
http://www.cbs.dtu.dk/services/SecretomeP/

9
http://www.stepdb.eu/MatureP.php

10
http://www.moonlightingproteins.org/

11
https://blast.ncbi.nlm.nih.gov/

## References

[B1] Adams M. D. Goglin K. Molyneaux N. Hujer K. M. Lavender H. Jamison J. J. (2008). Comparative genome sequence analysis of multidrug-resistant *Acinetobacter baumannii*. *J. Bacteriol.* 190 8053–8064. 10.1128/JB.00834-08 18931120 PMC2593238

[B2] Allen W. J. Phan G. Waksman G. (2012). Pilus biogenesis at the outer membrane of Gram-negative bacterial pathogens. *Curr. Opin. Struct. Biol.* 22 500–506. 10.1016/j.sbi.2012.02.001 22402496

[B3] Álvarez-Fraga L. Pérez A. Rumbo-Feal S. Merino M. Vallejo J. A. Ohneck E. J. (2016). Analysis of the role of the LH92_11085 gene of a biofilm hyper-producing *Acinetobacter baumannii* strain on biofilm formation and attachment to eukaryotic cells. *Virulence* 7 443–455. 10.1080/21505594.2016.1145335 26854744 PMC4871663

[B4] Antunes L. C. S. Visca P. Towner K. J. (2014). *Acinetobacter baumannii*: evolution of a global pathogen. *Pathog. Dis.* 71 292–301. 10.1111/2049-632X.12125 24376225

[B5] Aranda J. Poza M. Pardo B. G. Rumbo S. Rumbo C. Parreira J. R. (2010). A rapid and simple method for constructing stable mutants of *Acinetobacter baumannii*. *BMC Microbiol.* 10:279. 10.1186/1471-2180-10-279 21062436 PMC2993698

[B6] Aschtgen M. S. Bernard C. S. De Bentzmann S. Lloubès R. Cascales E. (2008). SciN is an outer membrane lipoprotein required for type VI secretion in enteroaggregative *Escherichia coli*. *J. Bacteriol.* 190 7523–7531. 10.1128/JB.00945-08 18805985 PMC2576670

[B7] Bao R. Nair M. K. M. Tang W. K. Esser L. Sadhukhan A. Holland R. L. (2013). Structural basis for the specific recognition of dual receptors by the homopolymeric pH 6 antigen (Psa) fimbriae of *Yersinia pestis*. *Proc. Natl. Acad. Sci. U.S.A.* 110 1065–1070. 10.1073/pnas.1212431110 23277582 PMC3549101

[B8] Basler M. Ho B. T. Mekalanos J. J. (2013). Tit-for-tat: type VI secretion system counterattack during bacterial cell-cell interactions. *Cell* 152 884–894. 10.1016/j.cell.2013.01.042 23415234 PMC3616380

[B9] Bendtsen J. D. Kiemer L. Fausbøll A. Brunak S. (2005a). Non-classical protein secretion in bacteria. *BMC Microbiol.* 5:58. 10.1186/1471-2180-5-58 16212653 PMC1266369

[B10] Bendtsen J. D. Nielsen H. von Heijne G. Brunak S. (2004). Improved prediction of signal peptides: signalp 3.0. *J. Mol. Biol.* 340 783–795. 10.1016/j.jmb.2004.05.028 15223320

[B11] Bendtsen J. D. Nielsen H. Widdick D. Palmer T. Brunak S. (2005b). Prediction of twin-arginine signal peptides. *BMC Bioinformatics* 6:167. 10.1186/1471-2105-6-167 15992409 PMC1182353

[B12] Bergogne-Bérézin E. Towner K. J. (1996). Acinetobacter spp. as nosocomial pathogens: microbiological, clinical, and epidemiological features. *Clin. Microbiol. Rev.* 9 148–165. 10.1128/cmr.9.2.148-165.1996 8964033 PMC172888

[B13] Berry A. A. Yang Y. Pakharukova N. Garnett J. A. Lee W. C. Cota E. (2014). Structural insight into host recognition by aggregative adherence fimbriae of enteroaggregative *Escherichia coli*. *PLoS Pathog.* 10:e1004404. 10.1371/journal.ppat.1004404 25232738 PMC4169507

[B14] Busch A. Waksman G. (2012). Chaperone-usher pathways: diversity and pilus assembly mechanism. *Philos. Trans. R. Soc. B Biol. Sci.* 367 1112–1122. 10.1098/rstb.2011.0206 22411982 PMC3297437

[B15] Carruthers M. D. Nicholson P. A. Tracy E. N. Munson R. S. (2013). *Acinetobacter baumannii* utilizes a type VI secretion system for bacterial competition. *PLoS One* 8:e59388. 10.1371/journal.pone.0059388 23527179 PMC3602014

[B16] Caselli E. Romagnoli C. Powers R. A. Taracila M. A. Bouza A. A. Swanson H. C. (2018). Inhibition of *Acinetobacter*-derived cephalosporinase: exploring the carboxylate recognition site using novel β-lactamase inhibitors. *ACS Infect. Dis.* 4 337–348. 10.1021/acsinfecdis.7b00153 29144725 PMC5987196

[B17] Chen C. Zabad S. Liu H. Wang W. Jeffery C. (2018). MoonProt 2.0: an expansion and update of the moonlighting proteins database. *Nucleic Acids Res.* 46 D640–D644. 10.1093/nar/gkx1043 29126295 PMC5753272

[B18] Chiu K. H. Wang L. H. Tsai T. T. Lei H. Y. Liao P. C. (2017). Secretomic analysis of host-pathogen interactions reveals that elongation factor-tu is a potential adherence factor of *Helicobacter pylori* during pathogenesis. *J. Proteome Res.* 16 264–273. 10.1021/acs.jproteome.6b00584 27764940

[B19] Choi C. H. Lee E. Y. Lee Y. C. Park T. I. Kim H. J. Hyun S. H. (2005). Outer membrane protein 38 of *Acinetobacter baumannii* localizes to the mitochondria and induces apoptosis of epithelial cells. *Cell. Microbiol.* 7 1127–1138. 10.1111/j.1462-5822.2005.00538.x 16008580

[B20] Choi C. H. Lee J. S. Lee Y. C. Park T. I. Lee J. C. (2008). *Acinetobacter baumannii* invades epithelial cells and outer membrane protein A mediates interactions with epithelial cells. *BMC Microbiol.* 8:216. 10.1186/1471-2180-8-216 19068136 PMC2615016

[B21] Choi K. H. Kumar A. Schweizer H. P. (2006). A 10-min method for preparation of highly electrocompetent *Pseudomonas aeruginosa* cells: application for DNA fragment transfer between chromosomes and plasmid transformation. *J. Microbiol. Methods* 64 391–397. 10.1016/j.mimet.2005.06.001 15987659

[B22] Choudhury D. Thompson A. Stojanoff V. Langermann S. Pinkner J. Hultgren S. J. (1999). X-ray structure of the FimC-FimH chaperone-adhesin complex from uropathogenic *Escherichia coli*. *Science* 285 1061–1066. 10.1126/science.285.5430.1061 10446051

[B23] Corcionivoschi N. Gundogdu O. Moran L. Kelly C. Scates P. Stef L. (2015). Virulence characteristics of hcp + *Campylobacter jejuni* and *Campylobacter coli* isolates from retail chicken. *Gut Pathog.* 7:20. 10.1186/s13099-015-0067-z 26207145 PMC4511981

[B24] Crouzet M. Claverol S. Lomenech A. M. Le Sénéchal C. Costaglioli P. Barthe C. (2017). *Pseudomonas aeruginosa* cells attached to a surface display a typical proteome early as 20 minutes of incubation. *PLoS One* 12:e0180341. 10.1371/journal.pone.0180341 28678862 PMC5498041

[B25] Crouzet M. Le Senechal C. Brözel V. S. Costaglioli P. Barthe C. Bonneu M. (2014). Exploring early steps in biofilm formation: set-up of an experimental system for molecular studies. *BMC Microbiol.* 14:253. 10.1186/s12866-014-0253-z 25266973 PMC4189659

[B26] Dallo S. F. Kannan T. R. Blaylock M. W. Baseman J. B. (2002). Elongation factor Tu and E1 β subunit of pyruvate dehydrogenase complex act as fibronectin binding proteins in *Mycoplasma pneumoniae*. *Mol. Microbiol.* 46 1041–1051. 10.1046/j.1365-2958.2002.03207.x 12421310

[B27] Das P. Lahiri A. Lahiri A. Chakravortty D. (2010). Modulation of the arginase pathway in the context of microbial pathogenesis: a metabolic enzyme moonlighting as an immune modulator. *PLoS Pathog.* 6:e1000899. 10.1371/journal.ppat.1000899 20585552 PMC2887468

[B28] Delano W. L. (2002). *The PyMOL Molecular Graphics System.* Available online at: https://ci.nii.ac.jp/naid/10025409089 (Accessed June 29, 2021).

[B29] Di Venanzio G. Flores-Mireles A. L. Calix J. J. Haurat M. F. Scott N. E. Palmer L. D. (2019). Urinary tract colonization is enhanced by a plasmid that regulates uropathogenic *Acinetobacter baumannii* chromosomal genes. *Nat. Commun.* 10:2763. 10.1038/s41467-019-10706-y 31235751 PMC6591400

[B30] Dijkshoorn L. Nemec A. Seifert H. (2007). An increasing threat in hospitals: multidrug-resistant *Acinetobacter baumannii*. *Nat. Rev. Microbiol.* 5 939–951. 10.1038/nrmicro1789 18007677

[B31] Dworkin J. (2015). Ser/Thr phosphorylation as a regulatory mechanism in bacteria. *Curr. Opin. Microbiol.* 24 47–52. 10.1016/j.mib.2015.01.005 25625314 PMC4380854

[B32] Fitzsimons T. C. Lewis J. M. Wright A. Kleifeld O. Schittenhelm R. B. Powell D. (2018). Identification of Novel *Acinetobacter baumannii* Type VI Secretion System Antibacterial Effector and Immunity Pairs. *Infect. Immun.* 86:e00297–18. 10.1128/IAI.00297-18 29735524 PMC6056853

[B33] Garnett J. A. Martínez-Santos V. I. Saldaña Z. Pape T. Hawthorne W. Chan J. (2012). Structural insights into the biogenesis and biofilm formation by the *Escherichia coli* common pilus. *Proc. Natl. Acad. Sci. U.S.A.* 109 3950–3955. 10.1073/pnas.1106733109 22355107 PMC3309717

[B34] Gaviard C. Cosette P. Jouenne T. Hardouin J. (2019). LasB and CbpD virulence factors of *Pseudomonas aeruginosa* carry multiple post-translational modifications on their lysine residues. *J. Proteome Res.* 18 923–933. 10.1021/acs.jproteome.8b00556 30672296

[B35] Gayoso C. M. Mateos J. Méndez J. A. Fernández-Puente P. Rumbo C. Tomás M. (2014). Molecular mechanisms involved in the response to desiccation stress and persistence in *acinetobacter baumannii*. *J. Proteome Res.* 13 460–476. 10.1021/pr400603f 24299215

[B36] Goic-Barisic I. Seruga Music M. Kovacic A. Tonkic M. Hrenovic J. (2017). Pan Drug-Resistant Environmental Isolate of *Acinetobacter baumannii* from *Croatia*. *Microb. Drug Resist.* 23 494–496. 10.1089/mdr.2016.0229 27792476

[B37] Hagemann L. Gründel A. Jacobs E. Dumke R. (2017). The surface-displayed chaperones GroEL and DnaK of *Mycoplasma pneumoniae* interact with human plasminogen and components of the extracellular matrix. *Pathog. Dis* 75:ftx017. 10.1093/femspd/ftx017 28204467

[B38] Harper T. M. June C. M. Taracila M. A. Bonomo R. A. Powers R. A. Leonard D. A. (2018). Multiple substitutions lead to increased loop flexibility and expanded specificity in *Acinetobacter baumannii* carbapenemase OXA-239. *Biochem. J.* 475 273–288. 10.1042/BCJ20170702 29229762 PMC5988212

[B39] Harvey K. L. Jarocki V. M. Charles I. G. Djordjevic S. P. (2019). The diverse functional roles of elongation factor Tu (EF-Tu) in microbial pathogenesis. *Front. Microbiol.* 10:2351. 10.3389/fmicb.2019.02351 31708880 PMC6822514

[B40] Henderson B. Martin A. (2013). Bacterial moonlighting proteins and bacterial virulence. *Curr. Top. Microbiol. Immunol.* 358 155–213. 10.1007/82_2011_188 22143554

[B41] Ho B. T. Fu Y. Dong T. G. Mekalanos J. J. (2017). *Vibrio cholerae* type 6 secretion system effector trafficking in target bacterial cells. *Proc. Natl. Acad. Sci. U.S.A.* 114 9427–9432. 10.1073/pnas.1711219114 28808000 PMC5584461

[B42] Hujer K. M. Hujer A. M. Hulten E. A. Bajaksouzian S. Adams J. M. Donskey C. J. (2006). Analysis of antibiotic resistance genes in multidrug-resistant Acinetobacter sp. isolates from military and civilian patients treated at the Walter Reed Army Medical Center. *Antimicrob. Agents Chemother.* 50 4114–4123. 10.1128/AAC.00778-06 17000742 PMC1694013

[B43] Jeffery C. (2018). Intracellular proteins moonlighting as bacterial adhesion factors. *AIMS Microbiol.* 4 362–376. 10.3934/microbiol.2018.2.362 31294221 PMC6604927

[B44] Jeffery C. (2019). The use of proteomics studies in identifying moonlighting proteins. *Methods Mol. Biol.* 1871 437–443. 10.1007/978-1-4939-8814-3_25 30276753

[B45] Jeffery C. J. (2018). Protein moonlighting: what is it, and why is it important? *Philos. Trans. R. Soc. B Biol. Sci.* 373:20160523. 10.1098/rstb.2016.0523 29203708 PMC5717523

[B46] Jeffery C. J. (2019). Intracellular/surface moonlighting proteins that aid in the attachment of gut microbiota to the host. *AIMS Microbiol.* 5 77–86. 10.3934/microbiol.2019.1.77 31384704 PMC6646928

[B47] Juncker A. S. Willenbrock H. von Heijne G. Brunak S. Nielsen H. Krogh A. (2003). Prediction of lipoprotein signal peptides in Gram-negative bacteria. *Protein Sci.* 12 1652–1662. 10.1110/ps.0303703 12876315 PMC2323952

[B48] Kang Q. Zhang D. (2020). Principle and potential applications of the non-classical protein secretory pathway in bacteria. *Appl. Microbiol. Biotechnol.* 104 953–965. 10.1007/s00253-019-10285-4 31853566

[B49] Karched M. Bhardwaj R. G. Tiss A. Asikainen S. (2019). Proteomic analysis and virulence assessment of granulicatella adiacens secretome. *Front. Cell. Infect. Microbiol.* 9:104. 10.3389/fcimb.2019.00104 31069174 PMC6491454

[B50] Kunert A. Losse J. Gruszin C. Hühn M. Kaendler K. Mikkat S. (2007). Immune evasion of the human pathogen *Pseudomonas aeruginosa*?: elongation factor tuf is a factor H and plasminogen binding protein. *J. Immunol.* 179 2979–2988. 10.4049/jimmunol.179.5.2979 17709513

[B51] Lai J. H. Yang J. T. Chern J. Chen T. L. Wu W. L. Liao J. H. (2016). Comparative phosphoproteomics reveals the role of AmpC β-lactamase phosphorylation in the clinical imipenem-resistant strain *Acinetobacter baumannii* SK17. *Mol. Cell. Proteomics* 15 12–25. 10.1074/mcp.M115.051052 26499836 PMC4762518

[B52] Lim C. L. L. Chua A. Q. Teo J. Q. M. Cai Y. Lee W. Kwa A. L. H. (2018). Importance of control groups when delineating antibiotic use as a risk factor for carbapenem resistance, extreme-drug resistance, and pan-drug resistance in *Acinetobacter baumannii* and *Pseudomonas aeruginosa*: a systematic review and meta-analysis. *Int. J. Infect. Dis.* 76 48–57. 10.1016/j.ijid.2018.05.017 29870795

[B53] Lipa P. Janczarek M. (2020). Phosphorylation systems in symbiotic nitrogen-fixing bacteria and their role in bacterial adaptation to various environmental stresses. *PeerJ* 8:e8466. 10.7717/peerj.8466 32095335 PMC7020829

[B54] Mijakovic I. Grangeasse C. Turgay K. (2016). Exploring the diversity of protein modifications: special bacterial phosphorylation systems. *FEMS Microbiol. Rev.* 40 398–417. 10.1093/femsre/fuw003 26926353

[B55] Moon K. H. Weber B. S. Feldman M. F. (2017). Subinhibitory concentrations of trimethoprim and sulfamethoxazole prevent biofilm formation by *Acinetobacter baumannii* through Inhibition of Csu Pilus expression. *Antimicrob. Agents Chemother.* 61 1–18. 10.1128/AAC.00778-17 28674047 PMC5571315

[B56] Mougous J. D. Cuff M. E. Raunser S. Shen A. Zhou M. Gifford C. A. (2006). A virulence locus of *Pseudomonas aeruginosa* encodes a protein secretion apparatus. *Science* 312 1526–1530. 10.1126/science.1128393 16763151 PMC2800167

[B57] N’Diaye A. R. Borrel V. Racine P. J. Clamens T. Depayras S. Maillot O. (2019). Mechanism of action of the moonlighting protein EfTu as a substance P sensor in *Bacillus cereus*. *Sci. Rep.* 9 1304. 10.1038/s41598-018-37506-6 30718605 PMC6361937

[B58] Nait Chabane Y. Ben Mlouka M. Alexandre S. Nicol M. Marti S. Pestel-Caron M. (2014). Virstatin inhibits biofilm formation and motility of *Acinetobacter baumannii*. *BMC Microbiol.* 14:62. 10.1186/1471-2180-14-62 24621315 PMC4007623

[B59] Orfanoudaki G. Markaki M. Chatzi K. Tsamardinos I. Economou A. (2017). MatureP: prediction of secreted proteins with exclusive information from their mature regions. *Sci. Rep.* 7:3263. 10.1038/s41598-017-03557-4 28607462 PMC5468347

[B60] Ouidir T. Jarnier F. Cosette P. Jouenne T. Hardouin J. (2014a). Extracellular Ser/Thr/Tyr phosphorylated proteins of *Pseudomonas aeruginosa* PA14 strain. *Proteomics* 14 2017–2030. 10.1002/pmic.201400190 24965220

[B61] Ouidir T. Jarnier F. Cosette P. Jouenne T. Hardouin J. (2014b). Potential of liquid-isoelectric-focusing protein fractionation to improve phosphoprotein characterization of *Pseudomonas aeruginosa* PA14. *Anal. Bioanal. Chem.* 406 6297–6309. 10.1007/s00216-014-8045-8 25096199

[B62] Pakharukova N. Garnett J. A. Tuittila M. Paavilainen S. Diallo M. Xu Y. (2015). Structural insight into archaic and alternative chaperone-usher pathways reveals a novel mechanism of pilus biogenesis. *PLoS Pathog.* 11:e1005269. 10.1371/journal.ppat.1005269 26587649 PMC4654587

[B63] Pakharukova N. Tuittila M. Paavilainen S. Malmi H. Parilova O. Teneberg S. (2018). Structural basis for *Acinetobacter baumannii* biofilm formation. *Proc. Natl. Acad. Sci. U.S.A.* 115 5558–5563. 10.1073/pnas.1800961115 29735695 PMC6003481

[B64] Parra-Millán R. Guerrero-Gómez D. Ayerbe-Algaba R. Pachón-Ibáñez M. E. Miranda-Vizuete A. Pachón J. (2018). Intracellular trafficking and persistence of *Acinetobacter baumannii* requires transcription factor EB. *mSphere* 3:e00106–18. 10.1128/msphere.00106-18 29600279 PMC5874439

[B65] Peleg A. Y. Seifert H. Paterson D. L. (2008). *Acinetobacter baumannii*: emergence of a successful pathogen. *Clin. Microbiol. Rev.* 21 538–582. 10.1128/CMR.00058-07 18625687 PMC2493088

[B66] Pukatzki S. McAuley S. B. Miyata S. T. (2009). The type VI secretion system: translocation of effectors and effector-domains. *Curr. Opin. Microbiol.* 12 11–17. 10.1016/j.mib.2008.11.010 19162533

[B67] Puorger C. Vetsch M. Wider G. Glockshuber R. (2011). Structure, folding and stability of FimA, the main structural subunit of type 1 Pili from uropathogenic *escherichia coli* strains. *J. Mol. Biol.* 412 520–535. 10.1016/j.jmb.2011.07.044 21816158

[B68] Rêgo A. T. Johnson J. G. Geibel S. Enguita F. J. Clegg S. Waksman G. (2012). Crystal structure of the MrkD1P receptor binding domain of *Klebsiella pneumoniae* and identification of the human collagen V binding interface. *Mol. Microbiol.* 86 882–893. 10.1111/mmi.12023 22988966

[B69] Remaut H. Rose R. J. Hannan T. J. Hultgren S. J. Radford S. E. Ashcroft A. E. (2006). Donor-strand exchange in chaperone-assisted pilus assembly proceeds through a concerted β strand displacement mechanism. *Mol. Cell* 22 831–842. 10.1016/j.molcel.2006.05.033 16793551 PMC7617774

[B70] Ruiz F. M. Santillana E. Spínola-Amilibia M. Torreira E. Culebras E. Romero A. (2015). Erratum: Crystal structure of Hcp from *Acinetobacter baumannii*: a component of the type VI secretion system. PLoS One 10, e0136978. 10.1371/journal.pone.0136978 26079269 PMC4469607

[B71] Rumbo-Feal S. Gómez M. J. Gayoso C. Álvarez-Fraga L. Cabral M. P. Aransay A. M. (2013). Whole transcriptome analysis of *Acinetobacter baumannii* assessed by RNA-Sequencing reveals different mRNA expression profiles in biofilm compared to planktonic cells. *PLoS One* 8:e72968. 10.1371/journal.pone.0072968 24023660 PMC3758355

[B72] Russell A. B. Hood R. D. Bui N. K. Leroux M. Vollmer W. Mougous J. D. (2011). Type VI secretion delivers bacteriolytic effectors to target cells. *Nature* 475 343–349. 10.1038/nature10244 21776080 PMC3146020

[B73] Sauer F. G. Fütterer K. Pinkner J. S. Dodson K. W. Hultgren S. J. Waksman G. (1999). Structural basis of chaperone function and pilus biogenesis. *Science* 285 1058–1061. 10.1126/science.285.5430.1058 10446050

[B74] Schneider C. A. Rasband W. S. Eliceiri K. W. (2012). NIH Image to ImageJ: 25 years of image analysis. *Nat. Methods* 9 671–675. 10.1038/nmeth.2089 22930834 PMC5554542

[B75] Schurtz Sebghati T. A. Korhonen T. K. Hornick D. B. Clegg S. (1998). Characterization of the type 3 fimbrial adhesins of Klebsiella strains. *Infect. Immun.* 66 2887–2894. 10.1128/iai.66.6.2887-2894.1998 9596764 PMC108286

[B76] Schwarz S. West T. E. Boyer F. Chiang W. C. Carl M. A. Hood R. D. (2010). Burkholderia type vi secretion systems have distinct roles in eukaryotic and bacterial cell interactions. *PLoS Pathog.* 6:e1001068. 10.1371/journal.ppat.1001068 20865170 PMC2928800

[B77] Sengstock D. M. Thyagarajan R. Apalara J. Mira A. Chopra T. Kaye K. S. (2010). Multidrug-resistant *Acinetobacter baumannii*: an emerging pathogen among older adults in community hospitals and nursing homes. *Clin. Infect. Dis.* 50 1611–1616. 10.1086/652759 20462357

[B78] Shrivastava S. R. Shrivastava P. S. Ramasamy J. (2018). World health organization releases global priority list of antibiotic-resistant bacteria to guide research, discovery, and development of new antibiotics. *J. Med. Soc.* 32 76–77. 10.4103/jms.jms_25_17 33692645

[B79] Silverman J. M. Agnello D. M. Zheng H. Andrews B. T. Li M. Catalano C. E. (2013). Haemolysin coregulated protein is an exported receptor and chaperone of type VI secretion substrates. *Mol. Cell* 51 584–593. 10.1016/j.molcel.2013.07.025 23954347 PMC3844553

[B80] Soares N. C. Cabral M. P. Gayoso C. Mallo S. Rodriguez-Velo P. Fernández-Moreira E. (2010). Associating growth-phase-related changes in the proteome of *Acinetobacter baumannii* with increased resistance to oxidative stress. *J. Proteome Res.* 9 1951–1964. 10.1021/pr901116r 20108952

[B81] Soares N. C. Spät P. Méndez J. A. Nakedi K. Aranda J. Bou G. (2014). Ser/Thr/Tyr phosphoproteome characterization of *Acinetobacter baumannii*: comparison between a reference strain and a highly invasive multidrug-resistant clinical isolate. *J. Proteomics* 102 113–124. 10.1016/j.jprot.2014.03.009 24657496

[B82] Standish A. J. Teh M. Y. Tran E. N. H. Doyle M. T. Baker P. J. Morona R. (2016). Unprecedented abundance of protein tyrosine phosphorylation modulates *Shigella flexneri* virulence. *J. Mol. Biol.* 428 4197–4208. 10.1016/j.jmb.2016.06.016 27380737

[B83] Stock A. M. Robinson V. L. Goudreau P. N. (2000). Two-component signal transduction. *Annu. Rev. Biochem.* 69 183–215. 10.1146/annurev.biochem.69.1.183 10966457

[B84] Sukumaran S. K. Fu N. Y. Tin C. B. Wan K. F. Lee S. S. Yu V. C. (2010). A soluble form of the pilus protein FimA targets the VDAC-hexokinase complex at mitochondria to suppress host cell apoptosis. *Mol. Cell* 37 768–783. 10.1016/j.molcel.2010.02.015 20347420

[B85] Tierney A. R. P. Rather P. N. (2019). Roles of two-component regulatory systems in antibiotic resistance. *Future Microbiol.* 14 533–552. 10.2217/fmb-2019-0002 31066586 PMC6526388

[B86] Tomaras A. P. Dorsey C. W. Edelmann R. E. Actis L. A. (2003). Attachment to and biofilm formation on abiotic surfaces by *Acinetobacter baumannii*: involvement of a novel chaperone-usher pili assembly system. *Microbiology* 149 3473–3484. 10.1099/mic.0.26541-0 14663080

[B87] Tucker A. T. Nowicki E. M. Boll J. M. Knauf G. A. Burdis N. C. Stephen Trent M. (2014). Defining gene-phenotype relationships in *acinetobacter baumannii* through one-step chromosomal gene inactivation. *mBio* 5 1–9. 10.1128/mBio.01313-14 25096877 PMC4128354

[B88] Vallenet D. Calteau A. Dubois M. Amours P. Bazin A. Beuvin M. (2020). MicroScope: an integrated platform for the annotation and exploration of microbial gene functions through genomic, pangenomic and metabolic comparative analysis. *Nucleic Acids Res.* 48 D579–D589. 10.1093/nar/gkz926 31647104 PMC7145621

[B89] Watanabe N. Osada H. (2012). Phosphorylation-dependent protein-protein interaction modules as potential molecular targets for cancer therapy. *Curr. Drug Targets* 13 1654–1658. 10.2174/138945012803530035 23030498

[B90] Weber B. S. Ly P. M. Irwin J. N. Pukatzki S. Feldman M. F. (2015). A multidrug resistance plasmid contains the molecular switch for type VI secretion in *Acinetobacter baumannii*. *Proc. Natl. Acad. Sci. U.S.A.* 112 9442–9447. 10.1073/pnas.1502966112 26170289 PMC4522760

[B91] Weber B. S. Miyata S. T. Iwashkiw J. A. Mortensen B. L. Skaar E. P. Pukatzki S. (2013). Genomic and functional analysis of the type VI secretion system in acinetobacter. *PLoS One* 8:e55142. 10.1371/journal.pone.0055142 23365692 PMC3554697

[B92] Werneburg G. T. Thanassi D. G. (2018). Pili assembled by the chaperone/usher pathway in *Escherichia coli* and *Salmonella*. *EcoSal. Plus* 8 10.1128/ecosalplus.esp-0007-2017 29536829 PMC5940347

[B93] Wood C. R. Ohneck E. J. Edelmann R. E. Actis L. A. (2018). A light-regulated type I pilus contributes to *Acinetobacter baumannii* biofilm, motility, and virulence functions. *Infect. Immun.* 86:e00442–18. 10.1128/IAI.00442-18 29891547 PMC6105899

[B94] Xie Y. Shao X. Zhang Y. Liu J. Wang T. Zhang W. (2019). *Pseudomonas* savastanoi two-component system RhpRS switches between virulence and metabolism by tuning phosphorylation state and sensing nutritional conditions. *MBio* 10:e02838-18. 10.1128/mBio.02838-18 30890603 PMC6426608

[B95] Yagüe P. Gonzalez-Quiñonez N. Fernánez-García G. Alonso-Fernández S. Manteca A. (2019). Goals and challenges in bacterial phosphoproteomics. *Int. J. Mol. Sci.* 20:9381. 10.3390/ijms20225678 31766156 PMC6888350

[B96] Yu N. Y. Wagner J. R. Laird M. R. Melli G. Rey S. Lo R. (2010). PSORTb 3.0: improved protein subcellular localization prediction with refined localization subcategories and predictive capabilities for all prokaryotes. *Bioinformatics* 26 1608–1615. 10.1093/bioinformatics/btq249 20472543 PMC2887053

[B97] Zavyalov V. Zavialov A. Zav’Yalova G. Korpela T. (2010). Adhesive organelles of Gram-negative pathogens assembled with the classical chaperone/usher machinery: structure and function from a clinical standpoint. *FEMS Microbiol. Rev.* 34 317–378. 10.1111/j.1574-6976.2009.00201.x 20070375

[B98] Ziveri J. Chhuon C. Jamet A. Rytter H. Prigent G. Tros F. (2019). Critical role of a sheath phosphorylation site on the assembly and function of an atypical Type VI secretion system. *Mol. Cell. Proteomics* 18 2418–2432. 10.1074/mcp.RA119.001532 31578219 PMC6885697

[B99] Zyła D. S. Prota A. E. Capitani G. Glockshuber R. (2019). Alternative folding to a monomer or homopolymer is a common feature of the type 1 pilus subunit FimA from enteroinvasive bacteria. *J. Biol. Chem.* 294 10553–10563. 10.1074/jbc.RA119.008610 31126987 PMC6615685

