# Supplementary material for: Phosphorylation of Extracellular Proteins in Acinetobacter baumannii in Sessile Mode of Growth
Source: Front Microbiol. 2021 Oct 1;12:738780. doi: 10.3389/fmicb.2021.738780 (PMC8517400; doi:10.3389/fmicb.2021.738780)
Supplement: Supplementary file 1 [file Data_Sheet_1.PDF]

Supplemental information – Materials and Methods

Phosphorylation of extracellular proteins of *Acinetobacter baumannii* in biofilm mode of growth

Sébastien Massier[a,b], Brandon Robin[a], Marianne Mégroz[c], Amy Wright[c], Marina Harper[c], Brooke Hayes[c] Pascal Cosette[a,b], Isabelle Broutin[d], John Boyce[c], Emmanuelle Dé[a], and Julie Hardouin[a,b,*]

[a] Normandy Univ, UNIROUEN, INSA Rouen, CNRS, Polymers, Biopolymers, Surface Laboratory, 76 000 Rouen, France
[b] PISSARO Proteomic Facility, IRIB, F-76820 Mont-Saint-Aignan, France
[c] Biomedicine Discovery Institute and Department of Microbiology, Monash University, Australia
[d] Université de Paris, CNRS, Laboratoire CiTCoM, 75006, Paris, France

Running title:
Extracellular phosphoproteins of *A. baumannii* biofilm

Extracellular protein digestion

Extracellular proteins were digested according to the following procedure. Proteins (50 µg) were mixed with 6X Laemmli buffer (58 mM Tris-HCl pH 6.8, 5% glycerol (v/v), 0.002% bromophenol blue (w/v), 0.1 M DTT, SDS 1.6% (w/v)), heated at 95°C for 5 min, and then loaded onto an SDS-PAGE stacking gel (7%). A short electrophoresis was performed (10 mA, 15 min). After migration, the gels were stained with Coomassie blue and destained with solution containing 50% ethanol, 10% acetic acid and 40% deionized water (v/v). Selected protein bands were excised, washed with water, alkylated with 15 mM iodoacetamide for 45 min in the dark before overnight digestion with trypsin (2 µg per band) with shaking. Peptide extraction was carried out 3 times with acetonitrile (ACN). Peptides were then dried completely using a Speedvac concentrator (SPD111V, Thermo Fisher Scientific) and stored at −20°C.

Phosphopeptide enrichment

Phosphopeptide enrichment was performed using metal oxide affinity chromatography (MOAC) with titanium dioxide beads (TiO_2, Carlo Erba) as previously described (20, 26). Before enrichment, the TiO_2 beads were washed first with ACN for 30 min and then with the loading buffer (ACN/H_2O (80/20), 0.1% trifluoroacetic acid (TFA) containing 5 mg/mL DHB) for 30 min with gentle mixing. The tryptic peptides were solubilized in the loading buffer, and then TiO_2 beads was added with a peptides: TiO_2 ratio of 1:10. The samples were incubated for 2 h with gentle mixing. After pelleting (2 min, 700 x g) the beads, the supernatant was transferred to the TiO_2 beads of the second enrichment step and the incubation and pelleting steps were repeated as above. The beads with the bound phosphopeptides were washed once with Wash solution I (ACN/H_2O (50/50), TFA 0.1%) and once with Wash solution II (ACN/H_2O (80/20), TFA 0.1%) for 5 min each, the phosphopeptides were then eluted with 0.5% ammonium hydroxide in ACN 80% (pH 11). After enrichment, peptides were desalted on C_{18} tip column (2 times), dried, and stored at -20°C until MS analysis. Three biological replicates were analysed.

NanoLC−MS/MS analyses

The enriched phosphopeptides were resuspended in 7 µL FA 1%, and 6 µL were injected either on the LTQ-Orbitrap Elite coupled to an Easy nLC II system or on the Qexactive Plus coupled to an Ultimate 3000 (all from Thermo Scientific).

For analyses on LTQ-Orbitrap Elite, samples were injected onto an enrichment column (C_{18} Acclaim PepMap100, Thermo Scientific) and the separation performed with an analytical column needle (NTCC-360/100-5-153, NikkyoTechnos). The mobile phase consisted of H_2O/FA 0.1% (buffer A) and ACN/FA 0.1% (buffer B). Tryptic peptides were eluted at a flow of 300 nL.min^{-1}, using a three-step linear gradient: from 2 to 40% B in 106 min, from 40 to 80% B in 4 min, and 80% B for 11 min. The mass spectrometer was operated in positive-ionization mode, with capillary voltage and source temperature set at 1.6 kV and 275 C, respectively. Phosphopeptides were analyzed using the neutral-loss-data-dependent MS^3 method. The first scan (MS spectra) was recorded in the Orbitrap analyzer ($R = 60{,}000$) with the mass range m/z 400–2000. Then, the 10 most intense ions were selected for MS^2 experiments. For phosphopeptide detection, MS^3 was initiated if a neutral-loss peak at either -26.6, –32.6, -39.9, or -49 relative to the precursor ion (corresponding to a neutral loss of phosphoric acid from doubly and triply-charged precursor ions) was identified in the MS^2 mass spectrum. Singly-charged species were excluded from MS(n) analysis. Dynamic exclusion of already fragmented precursor ions was applied for 30 s, with a repeat count of one, a repeat duration of 30 s, and an exclusion mass width of ±5 ppm. Fragmentation occurred in the linear ion-trap analyzer, with a collision energy of 35. All measurements in the Orbitrap analyzer were

performed with on-the-fly internal recalibration (lock mass) at *m/z* 445.12002 (polydimethylcyclosiloxane).

For analyses on Qexactive Plus, peptides were injected onto an enrichment column (C18 Pepmap100 precolumn (300 µm i.d. × 5 mm, 5 µm, 100 Å; Thermo Scientific). The separation was carried out using an EASY-spray column (15 cm, 3 µm, 0.075 mm, 100 Å; Thermo Scientific). The flow rate was set at 300 µL/min. The mobile phase was composed of H_2O/0.1% FA (buffer A) and ACN/H_2O/0.1% FA (80/20) (buffer B). The elution gradient duration was 120 min: 0-84 min, 2–35% B; 84-94 min, 35%-90% B; 94-105 min, 90% B; 105-120 min, 2% of B. The temperature of the column was set at 40°C. The maximum injection time was set to 100 ms. The capillary voltage was 1.6 kV. The temperature of the capillary was 275°C. The m/z detection range was 400–1800. The resolution was 70 000 in MS and 17 500 in MS/MS. The 10 most intense ions (Top 10) were selected and fragmented. Nitrogen was used as a collision gas and fragmentation occurred with a normalized collision energy of 27. All spectra obtained were exported in ".raw" format to identify peptides and proteins with Proteome Discoverer 1.4 software (Thermo Scientific). MS proteomics data have been deposited to the ProteomeXchange Consortium via the PRIDE partner repository with the data set identifier. Our project accession in PRIDE is PXD027208 (details for reviewers connexion: username : reviewer_pxd027208@ebi.ac.uk and password : eoWgokPC).

Database searches

Raw data files were processed using Proteome Discoverer 1.4 software (Thermo Scientific). Peak lists were searched using the MASCOT search software (Matrix Science) against the *A. baumannii* ATCC 17978 and *A. baumannii* AB0057 databases, containing 4097 and 4118 protein sequences respectively (http://www.genoscope.cns.fr). Database searches were performed with the following parameters: 2 missed trypsin cleavage sites allowed; variable modifications: carbamidomethylation on cystein, oxidation on methionine, and phosphorylation on serine/threonine/tyrosine. For analyses performed on LTQ-Orbitrap Elite, the parent-ion and daughter-ion tolerances were 5 ppm and 0.35 Da, respectively. For analyses performed on Qexactive Plus, the parent-ion and daughter-ion tolerances were 5 ppm and 0.05 Da, respectively. The false discovery rate (FDR) threshold for identifications was set at 1% (for proteins and peptides). For each identification, we considered a peptide ion score higher than 14 for phosphorylation; a peptide rank of 1, a q value and an expectation value below 0.05. To avoid biased automatic annotation, all phosphopeptide spectra were manually inspected. To validate a phosphorylated peptide, a minimal coverage of b- and y-fragment ion series was required: at least 5 successive daughter ions. For the localization of the phosphosites, the probabilities obtained from MASCOT and phosphoRS (Thermo Scientific) results were used.

Generation of an AB307-0294 *hcp* mutant and complementation strain

The AB307-0294 *hcp* mutant was generated as previously described (Adams et al., 2008; Aranda et al., 2010; Tucker et al., 2014) using splice overlap extension (SOE) PCR with the following modifications. Briefly, two 1.0-kb fragments that represented the region upstream and downstream of the *hcp* gene (ABBFA_02207) were amplified from AB307-0294 gDNA by PCR using primers BAP9130 and BAP9131 (Table S1) for the upstream region, or BAP9132 and BAP9133 for the downstream region. A third PCR fragment was generated that contained the kanamycin resistance gene (*neo*, ~1.0 kb) from pCR-BluntII-TOPO using primers BAP9134 and BAP9135. The primers for amplification of the kanamycin resistance gene included flanking Flp recombinase Recognition Target (FRT) sites (34 bp each) (Tucker et al., 2014) to allow for excision of the kanamycin resistance cassette via Flp recombinase; each also contained a 5' region (~30 bp) of complementarity to the AB307-0294 sequence upstream (BAP9134) or downstream (BAP9135) of the *hcp* gene. Each of the three PCRs were then

combined at equimolar concentrations and a second round SOE PCR performed using primers BAP9130 and BAP9133. For mutagenesis of *hcp*, the final SOE PCR product (∼3.0 kb) was introduced into the *A. baumannii* AB307-0294 strain via electroporation as previously described (Choi et al., 2006). Putative mutants were selected on lysogeny broth (LB) agar supplemented with kanamycin (50 µg/mL) and confirmed by PCR. To excise the kanamycin resistance gene, *neo*, the *hcp* mutant was transformed with pAT03 (conferring carbenicillin resistance), which expresses Flp recombinase; transformants were selected on LB agar supplemented with carbenicillin (100 µg/mL). To induce Flp recombinase expression, carbenicillin-resistant transformants were patched onto LB agar supplemented with carbenicillin (100 µg/mL) and isopropyl-β-d-1-thiogalactopyranoside (IPTG). Mutants with the *neo* gene excised were identified as Kan^s colonies by patching. To cure mutants of the pAT03 plasmid, a Kan^s colony was grown overnight in LB media at 37°C with shaking without addition of antibiotic. The culture was then plated onto LB agar and mutants lacking pAT03 identified by patching for $Carb^s/Kan^s$ colonies. The final Kan^s *hcp* mutant strain was confirmed by PCR and DNA sequencing using the genomic DNA as template and designated AL3831.
For complementation of the *hcp* mutant, an intact copy of the *hcp* gene including the native Ribosome binding site was amplified from WT AB307-0294 genomic DNA using primers BAP9140 and BAP9141, which contained BamHI and EcoRI restriction sites respectively. After digestion with BamHI and EcoRI, the amplified fragment was cloned into BamHI and EcoRI-digested pBASE (Table S2), such that the *A. baumannii* P_{tac} promoter would drive the transcription of *hcp*. The ligated products were then used to transform *E. coli* strain DH5α, before transformants containing the correct plasmid were identified by colony PCR using plasmid-specific primers Universal Primer and BAP8412. DNA sequencing was used to confirm the fidelity of the *hcp* sequence, and a correct recombinant plasmid was selected and designated pAL1686. The vector pBASE and the *hcp* complementation plasmid pAL1686 were then used to separately transform the AB307-0294 *hcp* mutant (AL3831) via electroporation, generating strains AL3844 (pBASE) and AL3942 (pAL1686).

<u>Mutation of individual Hcp phosphosites in *A. baumannii* strain AB307-0294</u>
For substitution of the Hcp S18 amino acid with alanine, the *hcp* gene was amplified by PCR using genomic DNA from wild-type *A. baumannii* strain AB307-0294 together with primers BAP9173 and BAP9141, which contained BamHI and EcoRI restriction sites. For substitution of the Hcp S18 amino acid with aspartic acid, the *hcp* gene was amplified by PCR using primers BAP9295 and BAP9141. SOE PCR was used for the individual Hcp S31, S41, T43 and S44 amino acid substitutions using primers listed in Table S1. The first SOE fragment was PCR-amplified using AB307-0294 genomic DNA as template and primer BAP9140 paired with either BAP9305 (for S31), BAP9307 (for S41), BAP9371 (for T43) or BAP9369 (for S44). The second SOE fragment was amplified with BAP9141 paired with either BAP9306 (for S31), BAP9308 (for S41), BAP9372 (for T43) or BAP9370 (for S44). Appropriate pairs of SOE PCR products were combined and amplified in a second round SOE PCR using BAP9140 together with BAP9141 and these PCR products were digested with BamHI and EcoRI and cloned into similarly-digested pBASE. The ligated products were then used to transform *E. coli* strain DH5α. *E. coli* transformants containing each of the correct plasmids were identified by colony PCR and confirmed by DNA sequencing; correct recombinant plasmids were selected and designated pAL1679 (S18A), pAL1743 (S18D), pAL1746 (S31A), pAL1747 (S41A), pAL1780 (T43A) or pAL1782 (S44A). The modified-*hcp* complementation plasmids were then used to separately transform the AB307-0294 *hcp* mutant (AL3831) via electroporation, generating strains AL3895 (pAL1679), AL4101 (pAL1743), AL4104 (pAL1746), AL4105 (pAL1747), AL4176 (pAL1780) and AL4178 (pAL1782) (Table S2).

Detection of the T6SS protein Hcp using western immunoblotting

For the detection of the Hcp protein in *A. baumannii* whole-cell lysates (WCL) and culture supernatants using western immunoblotting, protein fractions were prepared and examined as described previously (Fitzsimons et al., 2018), with the following modifications. Briefly, AB307-0294 strains were grown to late-exponential growth phase (OD_{600} = 1.5) in LB medium with addition of antibiotics as required. To prepare culture supernatants, 350 µL of bacterial culture was centrifuged at 11,000 x g for 5 min. A volume of supernatant was removed and mixed at a 1:1 volume ratio with 2 x SDS-PAGE sample buffer. The cell pellet was washed once with phosphate-buffered saline (PBS; pH 7.4), resuspended in 35 µL of PBS and mixed at a 1:1 volume ratio with 2 x SDS-PAGE sample buffer to prepare a 10 x concentrated WCL sample. All samples were boiled for 10 min and WCL samples centrifuged at 11, 000 x g for 5 min. Samples were separated on a 17.5% acrylamide gel using SDS-PAGE and then transferred to polyvinylidene fluoride (PVDF) (Merck, Millipore) membranes via electroblotting. Hcp was detected using anti-Hcp antiserum (Fitzsimons et al., 2018) followed by the secondary antibody, donkey anti-chicken horseradish peroxidase (HRP) conjugate, each at a dilution of 1:500. The Clarity™ Western ECL Substrate was used to detect antibody binding and membranes visualised using the Amersham Imager 680 (GE Healthcare Life Sciences).

Band intensity was quantified using ImageJ software (Schneider et al., 2012), with the "Subtract Background" tool, using the rolling ball radius method (ball radius value of 50.0 pixels). Bands were selected followed by quantification of peak area of obtained histograms. To determine if any of the differences in Hcp protein production detected in *A. baumannii* protein fractions via western immunoblotting were statistically significant, one-way analysis of variance (ANOVA) was employed (GraphPad Prism, version 9) with Turkey's multiple-comparison post-test (GraphPad Prism); a *P* value of <0.05 was accepted as statistically significant.
