# Supplementary material for: Phosphorylation of Extracellular Proteins in Acinetobacter baumannii in Sessile Mode of Growth
Source: Front Microbiol. 2021 Oct 1;12:738780. doi: 10.3389/fmicb.2021.738780 (PMC8517400; doi:10.3389/fmicb.2021.738780)
Supplement: Supplementary file 2 [file Data_Sheet_2.PDF]

Supplementary Figures

Phosphorylation of extracellular proteins of *Acinetobacter baumannii* in biofilm mode of growth

Sébastien Massier[a,b], Brandon Robin[a], Marianne Mégroz[c], Amy Wright[c], Marina Harper[c], Brooke Hayes[c] Pascal Cosette[a,b], Isabelle Broutin[d], John Boyce[c], Emmanuelle Dé[a], and Julie Hardouin[a,b,*]

[a] Normandy Univ, UNIROUEN, INSA Rouen, CNRS, Polymers, Biopolymers, Surface Laboratory, 76 000 Rouen, France
[b] PISSARO Proteomic Facility, IRIB, F-76820 Mont-Saint-Aignan, France
[c] Biomedicine Discovery Institute and Department of Microbiology, Monash University, Australia
[d] Université de Paris, CNRS, Laboratoire CiTCoM, 75006, Paris, France

Running title:
Extracellular phosphoproteins of *A. baumannii* biofilm

Figure S1

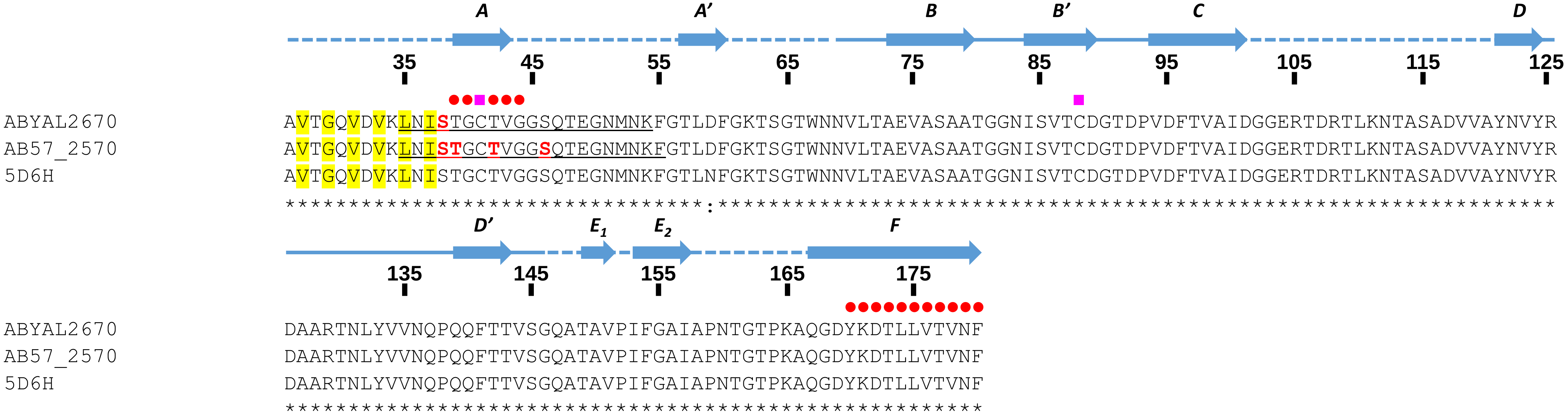

<u>Figure S1</u>: Alignment of major fimbrial subunits CsuA/B in A. *baumannii* AB0057 and ATCC 17978.
Alignment of major fimbrial subunits CsuA/B in *A. baumannii* AB0057 and ATCC 17978.
Alignment of major fimbrial subunits CsuA/B in ATCC 17978 (ABYAL2670), AB0057 (AB57_2570) with the sequence of CsuA/B available in the PDB database (5D6H). Pilin N-terminal residues in the protein proposed to take part in donor strand complementation are indicated in yellow, residues involved in interaction with the A1 and G1 β-strands of the chaperone CsuC are indicated by red circles. Completely invariant residues are indicated with an asterisk (*), highly conserved residues are indicated with a colon (:) and semi-conserved residues are indicated with a dot (.). Conserved cysteine residues involved in the disulfide bridge are indicated by pink squares. Identified peptides and phospho-sites in our study are underlined and colored in red (and bold), respectively. Limits and nomenclature for secondary structure elements are shown above the sequence. β-Strands are depicted by arrows above the alignments. Dashed line indicates unstructured regions. In this alignment, peptide signal sequences were removed. Residues S36 in AB57_2570 and S38 in ABYAL2670 are represented by S13 in this alignment and I35 corresponds to I12.

Figure S2

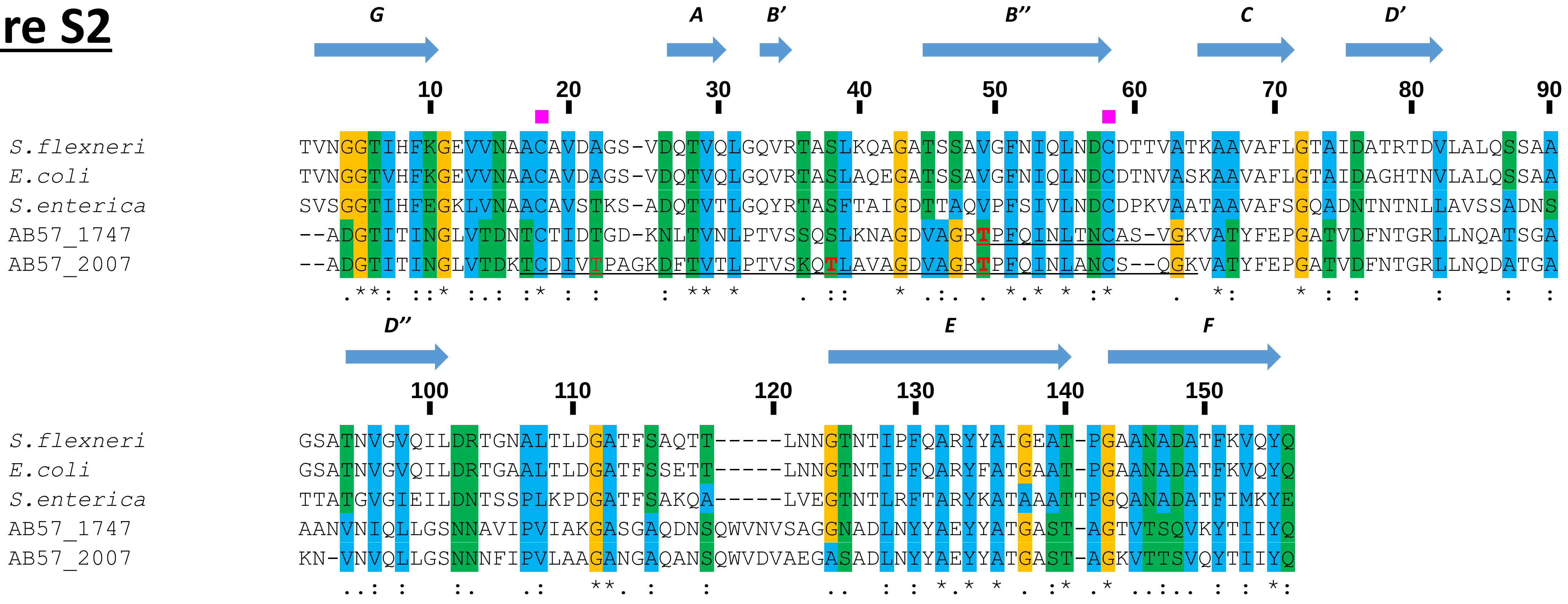

<u>Figure S2</u>: Alignment of FimA-like main pilin from A. baumannii AB0057 (AB57_1747 and AB57_2007) with FimA proteins from different species (available in the PDB database) such as S. flexneri (5LP9), E. coli (5NTK) and S. enterica (6ERJ).
Conserved cysteine residues involved in the disulfide bridge are indicated by pink squares.
Completely invariant residues are indicated with an asterisk (*), highly conserved residues are indicated with a colon (:) and semi-conserved residues are indicated with a dot (.)highly conserved regions are marked with a colon (:) and residues essential for semi-conservation are dotted (.). Residues are coded as follows: Color-coding scheme of amino acid residues has been incorporated above conservation threshold according to their chemical properties: blue – hydrophobic, green – hydrophilic and orange – neither hydrophobic nor hydrophilic (glycine residues). Identified peptides and phospho-sites in our study are respectively underlined and colored in red (and in bold), respectively. Limits and nomenclature for secondary structure elements are shown above the sequence. β-Strands are depicted by arrows above the alignments.

Figure S3

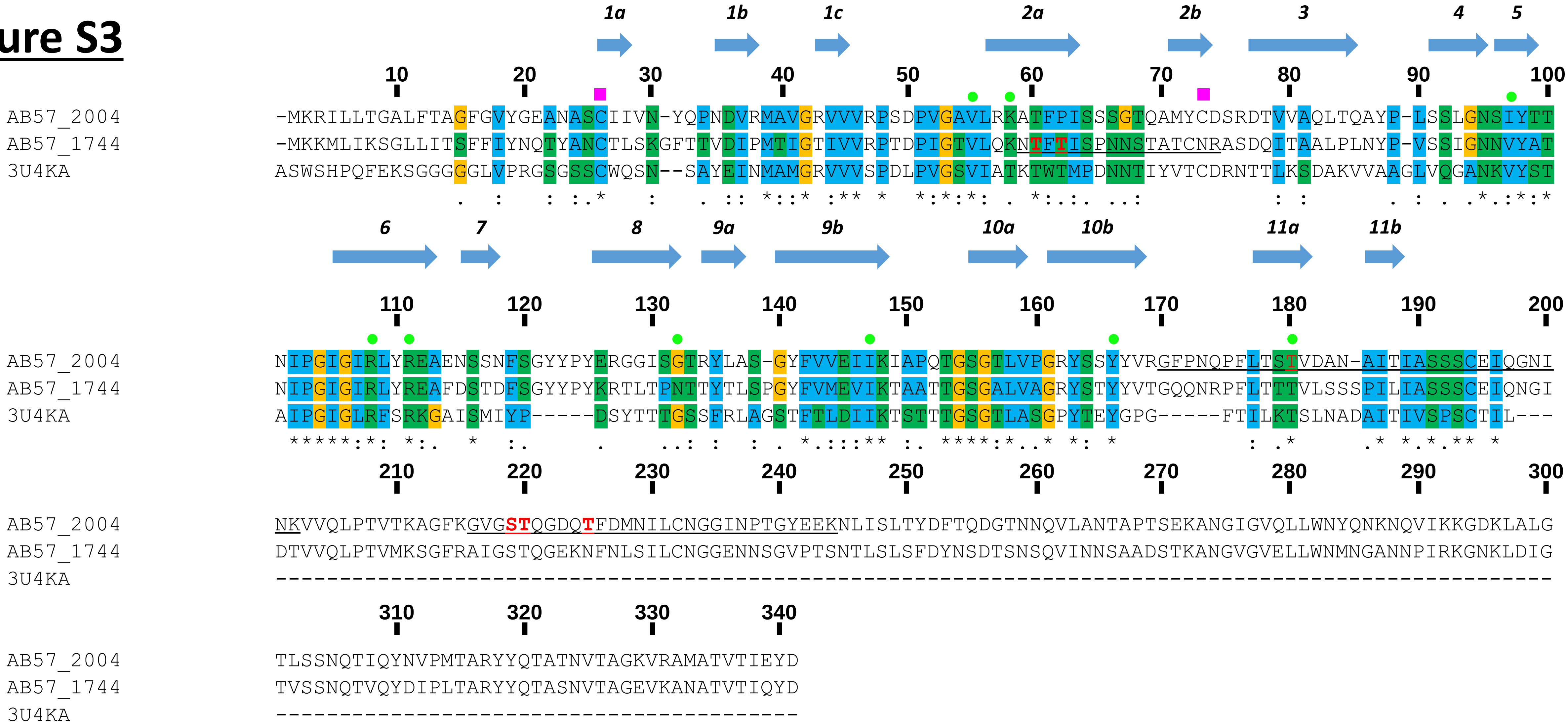

Figure S3: **Alignment of tip pilins from A. *baumannii* AB0057 (AB57_1744 and AB57_2004) with the adhesin MrkD of *Klebsiella pneumoniae* (3U4KA in PDB).** The important residues involved in collagen V binding are indicated by green circles. Completely invariant residues are indicated with an asterisk (*), highly conserved residues are indicated with a colon (:) and semi-conserved residues are indicated with a dot (.). Color-coding scheme of amino acid residues has been incorporated above conservation threshold according to their chemical properties: blue – hydrophobic, green – hydrophilic and orange – neither hydrophobic nor hydrophilic (glycine residues). Conserved cysteine residues involved in the disulfide bridge are indicated by pink squares. Identified peptides and phospho-sites in our study are underlined and colored in red (and in bold), respectively. Limits and nomenclature for secondary structure elements are shown above the sequence. β-Strands are depicted by arrows above the alignments.
