# Supplementary material for: Phosphorylation of Extracellular Proteins in Acinetobacter baumannii in Sessile Mode of Growth
Source: Front Microbiol. 2021 Oct 1;12:738780. doi: 10.3389/fmicb.2021.738780 (PMC8517400; doi:10.3389/fmicb.2021.738780)
Supplement: Supplementary file 3 [file Data_Sheet_3.pdf]

Table S1. Primers used in this study.

Primer	Sequence (5′ – 3′)	Description
BAP8412	AAAAACCATGGCAGGTCGTAAATCACTGCATAATTCG	Reverse primer for PCR/sequencing of DNA inserted into pBASE; binds downstream of MCS
BAP9130	CTTGAGCAAATCTCTCATGTTGC	Forward primer for upstream fragment of *hcp* mutagenesis construct, used to generate *hcp* deletion mutant AL3831
BAP9131	CGTATATATCTTTCATTTAGAACTTTCCT	Reverse primer for upstream fragment of *hcp* mutagenesis construct, used to generate *hcp* deletion mutant AL3831. The 5' end is complementary to the sequence at the 5' end of the Kan^r cassette
BAP9132	GCTTCTTACGCAGCGTAATTATTTAAG	Forward primer for downstream fragment of *hcp* mutagenesis construct, used to generate *hcp* deletion mutant AL3831. The 5' end is complementary to the sequence at the 3' end of the Kan^r cassette
BAP9133	ATTAAACTCACACTGCACCCCTTTG	Reverse primer for downstream fragment of *hcp* mutagenesis construct, used to generate *hcp* deletion mutant AL3831
BAP9134	AGGAAAGTTCTAAATGAAAGATATATACGGAAGTTCCTATACTTTCTAGAGAATAGGAACTTCCCGGAATTGCCA	Forward primer for amplification of the kanamycin resistance cassette from pCR-BluntII-TOPO, used to generate *hcp* deletion mutant AL3831. The 5' end contains FRT sequence and region complementary to BAP9131
BAP9135	CTTAAATAATTACGCTGCGTAAGAAGCGAAGTTCCTATTCTCTAGAAAGTATAGGAACTTCTTCAGAAGAACTCG	Reverse primer for amplification of the kanamycin resistance cassette from pCR-BluntII-TOPO, used to generate *hcp* deletion mutant AL3831. The 5' end contains FRT sequence and region complementary to BAP9132
BAP9140	AAAAAAGGATCCAGGAAAGTTCTAAATGAAAGATATATACG	Forward primer for amplification of AB307-0294 *hcp* (ABBFA_02207); contains native *hcp* RBS and BamHI site

BAP9141	AAAAAAGAATTCCTTAAATAATT ACGCTGCGTAAGAAGC	Reverse primer for amplification of AB307-0294 *hcp*; contains EcoRI
BAP9173	AAAAAAGGATCCAGGAAAGTTCT AAATGAAAGATATATACGTTGAG TTTCGCGGTAAATATAAAGTTGAT GGAGAGGCTCGTGATTCTGAG	Forward primer for amplification of AB307-0294 *hcp* with a Ser18Ala (S18A) amino acid substitution; contains native *hcp* RBS and BamHI site
BAP9295	AAAAAAGGATCCAGGAAAGTTCT AAATGAAAGATATATACGTTGAG TTTCGCGGTAAATATAAAGTTGAT GGAGAGGATCGTGATTCTGAG	Forward primer for amplification of AB307-0294 *hcp* with a Ser18Asp (S18D) amino acid substitution; contains native *hcp* RBS and BamHI site
BAP9305	GGATGTTATGAGACCAAGCGTTA ACTTCTAACC	Reverse primer for use in combination with BAP9140 for amplification of AB307-0294 *hcp* with a Ser31Ala (S31A) amino acid substitution; to generate product for SOE PCR reaction
BAP9306	GGTTAGAAGTTAACGCTTGGTCTC ATAACATCC	Forward primer for use in combination with BAP1941 for amplification of AB307-0294 *hcp* with a Ser31Ala (S31A) amino acid substitution; to generate product for SOE PCR reaction
BAP9307	CCACACTACTTGAAGTAGCAGCTT TAGGTTGAC	Reverse primer for use in combination with BAP9140 for amplification of AB307-0294 *hcp* with a Ser41Ala (S41A) amino acid substitution; to generate product for SOE PCR reaction
BAP9308	CGTCAACCTAAAGCTGCTACTTCA AGTAGTGTG	Forward primer for use in combination with BAP9141 for amplification of AB307-0294 *hcp* with a Ser41Ala (S41A) amino acid substitution; to generate product for SOE PCR reaction
BAP9369	CCCACACTACTTGCAGTAGCAGAT TTAGG	Reverse primer for use in combination with BAP9140 for amplification of AB307-0294 *hcp* with a Ser44Ala (S44A) amino acid substitution; to generate product for SOE PCR reaction
BAP9370	CCTAAATCTGCTACTGCAAGTAGT GTGGG	Forward primer for use in combination with BAP9141 for amplification of AB307-0294 *hcp* with a Ser44Ala (S44A) amino acid

		substitution; to generate product for SOE PCR reaction
BAP9371	CCCACACTACTTGAAGCAGCAGATTTAGG	Reverse primer for use in combination with BAP9140 for amplification of AB307-0294 *hcp* with a Thr43Ala (T43A) amino acid substitution; to generate product for SOE PCR reaction
BAP9372	CCTAAATCTGCTGCTTCAAGTAGTGTGGG	Forward primer for use in combination with BAP9141 for amplification of AB307-0294 *hcp* with a Thr43Ala (T43A) amino acid substitution; to generate product for SOE PCR reaction
Universal Primer (BAP8807)	GTAAAACGACGGCCAGT	Forward primer for PCR/sequencing of DNA inserted into pBASE; binds upstream of MCS

Table S2. Bacterial strains and plasmids used in this study.

Strain or plasmid	Relevant description	Source or reference
Strains		
E. coli strains		
DH5α	*deoR endA1 gyrA96 hsdR17*(r_K^- m_K^+) *recA1 relA1 supE44 thi-1* (*lacZYA-argFV169*) φ80*lacZ*ΔM15, F^-	Bethesda Research Laboratories
A. baumannii strains		
AB307-0294	*A. baumannii* AB307-0294 wild-type, clinical isolate	Adams et al., 2008
AL2730	AB307-0294 derivative with *tssM* replaced by allelic exchange with a kanamycin resistance cassette; Kan^r; Δ*tssM*	Fitzsimons et al 2018
AL3831	AB307-0294 *hcp* (ABBFA_02207) deletion mutant with a 34 bp FRT scar at *hcp* excision site; Δ*hcp*	This study
AL3844	AL3831 harboring pBASE as a complementation control*;* $Carb^r$	This study
AL3895	AL3831 complemented with pAL1679; $Carb^r$; S18A	This study
AL3942	AL3831 complemented with pAL1686; $Carb^r$; *hcp*	This study
AL4101	AL3831 complemented with pAL1743; $Carb^r$; S18D	This study
AL4104	AL3831 complemented with pAL1746; $Carb^r$; S31A	This study
AL4105	AL3831 complemented with pAL1747; $Carb^r$; S41A	This study
AL4176	AL3831 complemented with pAL1780; $Carb^r$; T43A	This study
AL4178	AL3831 complemented with pAL1782; $Carb^r$; S44A	This study
Plasmids		
pAT03	Broad host range plasmid encoding FLP recombinase; Amp^r/$Carb^r$	Tucker et al., 2014
pAL1679	pBASE containing *hcp* with a Ser18Ala (S18A) amino acid substitution; amplified from AB307-0294 using primers BAP9173 and BAP9141 and cloned into BamHI and EcoRI sites; Amp^r/$Carb^r$	This study
pAL1686	pBASE containing wild-type *hcp*, amplified from AB307-0294 using primers BAP9140	This study

	and BAP9141 and cloned into BamHI and EcoRI sites; contains native RBS; $Amp^r/Carb^r$	
pAL1743	pBASE containing *hcp* with a Ser18Asp (S18D) amino acid substitution; amplified from AB307-0294 using primers BAP9295 and BAP9141 and cloned into BamHI and EcoRI sites; $Amp^r/Carb^r$	This study
pAL1746	pBASE containing *hcp* with Ser31Ala (S31A) amino acid substitution; amplified from AB307-0294 using SOE PCR with primers BAP9140/BAP9305 and BAP9306/BAP9141 and cloned into BamHI and EcoRI sites; $Amp^r/Carb^r$	This study
pAL1747	pBASE containing *hcp* with Ser41Ala (S41A) amino acid substitution; amplified from AB307-0294 using SOE PCR with primers BAP9140/BAP9307 and BAP9308/BAP9141 and cloned into BamHI and EcoRI sites; $Amp^r/Carb^r$	This study
pAL1780	pBASE containing *hcp* with Thr43Ala (T43A) amino acid substitution; amplified from AB307-0294 using SOE PCR with primers BAP9140/BAP9371 and BAP9372/BAP9141 and cloned into BamHI and EcoRI sites; $Amp^r/Carb^r$	This study
pAL1782	pBASE containing *hcp* with Ser44Ala (S44A) amino acid substitution; amplified from AB307-0294 using SOE PCR with primers BAP9140/BAP9369 and BAP9370/BAP9141 and cloned into BamHI and EcoRI sites; $Amp^r/Carb^r$	This study
pBASE	*A. baumannii-E. coli* shuttle and protein expression vector; contains the P_{tac} promoter, $Amp^r/Carb^r$	Fitzsimons et al 2018
pCR-BluntII-TOPO	Suicide vector for *A. baumannii*; Kan^r/Zeo^r	Invitrogen
